# Chromosome 22q13 terminal deletion size is associated with relevant clinical features in a sample of 63 Italian patients with Phelan-McDermid syndrome

**DOI:** 10.1186/s11689-026-09708-x

**Published:** 2026-05-26

**Authors:** Laura Sandoni, Fethia Chehbani, Lisa Asta, Michela Camia, Arianna Ricciardello, Pasquale Tomaiuolo, Francesca Cucinotta, Laura Turriziani, Maria Boncoddo, Fabiana Bellomo, Marco Baccarin, Chiara Picinelli, Paola Castronovo, Roberto Sacco, Carla Lintas, Ignazio Stefano Piras, Francesco Pelagatti, Federico Banchelli, Riccardo Cuoghi Costantini, Roberto D’Amico, Antonio M. Persico

**Affiliations:** 1 Department of Biomedical, Metabolic and Neural Sciences, University of Modena and Reggio Emilia, Modena, Italy; 2 Cantonal Psychiatric Clinic, Cantonal Socio-Psychiatric Organization (O.S.C.), Repubblica E Cantone Ticino, Mendrisio, Switzerland; 3 Mafalda Luce Center for Pervasive Developmental Disorders, Milan, Italy; 4 IRCCS Centro Neurolesi ”Bonino-Pulejo”, Messina, Italy; 5 Center for Autism “Dopo Di Noi”, Barcellona Pozzo Di Gotto (Messina), Italy; 6 Institute for Biomedical Research and Innovation (IRIB), National Research Council (CNR), Messina, Italy; 7 Child Neuropsychiatry Unit, “G. Martino” University Hospital, Messina, Italy; 8 Medical Genetics Service (EOLAB), Ente Ospedaliero Cantonale, Bellinzona, Switzerland; 9 Service for Neurodevelopmental Disorders & Laboratory of Molecular Psychiatry and Neurogenetics, University “Campus Bio-Medico”, Rome, Italy; 10 Research Unit of Medical Genetics, Department of Medicine, University “Campus Bio-Medico”, Rome, I-00128 Italy; 11 Operative Research Unit of Medical Genetics, Fondazione Policlinico Universitario Campus Bio-Medico, Rome, I-00128 Italy; 12 Division of Early Detection and Prevention, The Translational Genomics Research Institute, Phoenix, AZ 85004 USA; 13 Unit of Statistical and Methodological Support to Clinical Research, Modena Univ. Hospital, Modena, Italy; 14 Department of Medical and Surgical Sciences, University of Modena and Reggio Emilia, Modena, Italy; 15 Child & Adolescent Neuropsychiatry Program, Modena University Hospital, Modena, Italy

**Keywords:** Autism, Chromosome 22q13.3 deletion syndrome, Genetic association studies, Genotype–phenotype correlation, Intellectual disability, Phelan-McDermid syndrome, *SHANK3*, Telomeric 22q13 monosomy syndrome

## Abstract

**Background:**

Phelan-McDermid syndrome (PMS) is caused in the majority of cases by the loss or mutation of one allele of the *SHANK3* gene, located in human chr 22q13.33. PMS displays large interindividual differences in clinical severity and longitudinal trajectory. Other genes located in this chromosomal region are known to contribute to the clinical phenotype (*CELSR1*, *TCF20*) in patients with larger deletions. The aim of this study is to identify clinically-relevant phenotypic features significantly influenced by the size of chromosome 22q terminal deletion and to identify new potential candidate genes likely to be involved in these phenotypic effects.

**Methods:**

Genotype–phenotype correlations were investigated in 63 PMS patients directly ascertained by deep clinical phenotyping and determination of deletion size (Agilent CGH-array 180K or 400K). Patients were partitioned into eleven categories, based on deletion size (Mb). Phenotypic variables significantly influenced by deletion size were initially detected by exact χ^2^ (10,000 iterations) and Kendall’s Tau. Candidate genes were then sought using: (a) ROC curves for binary dichotomous variables; (b) best separation threshold for quantitative variables.

**Results:**

Phenotypic variables significantly associated with chromosome 22q deletion size in our sample include: expressive language (*p* < 0.001); motor development timing (*p* < 0.001); gait (*p* < 0.001); muscle strength (*p* < 0.01); social cognition, encompassing eye contact, exchange gesture, and joint attention (*p* < 0.001—< 0.05); infectious diseases coincident with the onset of behavioral manifestations (*p* < 0.001); brain structural abnormalities on MRI (*p* < 0.001); dysmorphisms (*p* < 0.001); renal and urinary malformations (*p* < 0.01); comorbid lifelong bipolar disorder (*p* < 0.05). The best separation thresholds for many of these variables were located within or nearby genes playing important morphogenetic (*PLXNB2, TAFA5*) or neurodevelopmental roles (*BRD1*, *TBC1D22A*, *ATXN10* and/or *FBLN1*). For renal malformations, the two best thresholds point toward one long non-coding RNA and a cluster of antisense RNAs.

**Conclusions:**

The genes identified in this study appear as strong candidates to contribute to the PMS phenotype, by conferring an additional layer of abnormal neurodevelopment and impaired morphogenesis to the disruptive effects produced by *SHANK3* haploinsufficiency.

**Supplementary Information:**

The online version contains supplementary material available at https://doi.org/10.1186/s11689-026-09708-x.

## Introduction

Phelan-McDermid syndrome (PMS), also known as “chromosome 22q13.3 deletion syndrome” (OMIM #606,232), is a rare genetic disorder mainly characterized by global developmental delay and/or intellectual disability, muscle hypotonia, severely impaired or absent speech, and minor/major dysmorphisms [1–3]. Patients may also present several medical conditions, including abnormal EEG (with or without seizures) [4–6], structural brain anomalies at the MRI [7–10], sleep disturbances [11, 12], renal malformations, gastroesophageal reflux, lymphedema [13–15], and immune deficits [3, 14, 16]. The exact prevalence of PMS is still unknown. Currently, over 3,600 PMS individuals worldwide are registered in the PMS Foundation Registry [17]; however, the syndrome is likely underdiagnosed, given its nonspecific clinical phenotype and the need for genetic testing [18, 19].

The most frequent cause of PMS is represented by terminal deletions of chromosome 22q13.3 involving the *SHANK3* gene, in a minority of cases due to ring chromosome 22 or to unbalanced translocations, followed by *SHANK3* intragenic deletions and pathogenic mutations [1, 2, 4, 20]. *SHANK3* encodes for a scaffolding protein abounding in the postsynaptic density (PSD) of glutamatergic synapses and is involved in dendritic spines and synapse formation [21]. Within the framework of PMS, in addition to global developmental delay and intellectual disability, *SHANK3* haploinsufficiency promotes other frequently co-occurring behavioral conditions, like bipolar disorder [22–25], catatonia [24–27], and autism spectrum disorder (ASD) [28–33], with a diagnosis of ASD sometimes preceding the results of genetic testing by several years in clinical practice. Also *SHANK3* mutations represent one of the most common monogenic cause of autism, especially in cases with moderate to profound intellectual disability [31, 34, 35]. The latter observation, coupled with the fact that usually even the smallest pathogenic deletions at least partly span *SHANK3* [7, 14, 19], has long proven its pathogenic role in the PMS phenotype [9, 20]. However, interstitial chromosome 22q deletions sparing *SHANK3* have also been found associated with PMS, albeit occasionally [16, 36]. This rare but important finding further underscores the phenotypic roles of additional genes centromeric to *SHANK3* in the chromosome 22q region, and has led to propose a distinction between “*SHANK3*-related” and “*SHANK3*-unrelated” PMS [37].

Great interindividual heterogeneity can be clinically observed among PMS patients. This heterogeneity may at least partly be explained by phenotypic contributions by 22q13 genes other than *SHANK3* [36–39]. Deletion size can vary from less than 100 kb to > 9 Mb in PMS [16, 40], and genotype–phenotype studies indicate that larger deletions generally lead to more severe symptomatology [41]. Larger deletions have been associated with severity of developmental delay, including absent or impaired speech and walking ability [16, 19, 40, 42, 43]; abnormal growth, including macrocephaly and large hands [16, 42]; neonatal hypotonia [40, 43]; dysmorphic features [3, 16, 40]; number of medical comorbidities [3]; seizures [40]; adaptive skills [44]; renal abnormalities and lymphedema [14, 40]; congenital heart defects, neuroimaging brain anomalies and recurrent infections [40]. Some studies have attempted to precisely identify chromosome 22q13 genomic regions or candidate genes responsible for specific clinical features in PMS. For instance, Sarasua and Colleagues [43] identified genomic regions significantly associated with absent speech (boundaries: 40.4—49.4 Mb, median deletion size: 7.0 Mb), delayed speech (boundaries: 44.4—49.5, median deletion size: 3.3 Mb), macrocephaly, tall stature, and large or flashy hands (boundaries 44.5—46.6 Mb). Interestingly, the genomic region spanning 41.9 to 46.6 Mb was associated with decreased prevalence of autism and aggressive behavior [43]. To date the gene with the best established role in PMS besides *SHANK3* is *TCF20*, responsible for an autosomal dominant form of intellectual disability, with developmental delay, muscle hypotonia, variable dysmorphic features, movement disorders, and sleep disturbances [45]. This gene, however, is included only in the largest terminal deletions found in a small minority of PMS patients (see Results). *CELSR1* is another gene located on chromosome 22q13 whose haploinsufficiency has been demonstrated significantly associated with the risk to develop lymphedema in PMS [13].

The purpose of the present study is to move beyond the well-established role of *SHANK3* in PMS and to investigate the contributions provided by other genes to a set of clinically relevant phenotypic features in a sample of 63 directly-ascertained Italian PMS patients carrying 22q13 deletions of various sizes. Understanding the impact of deletion size and related genes on the clinical phenotype and on the developmental trajectory of PMS patients is important, because it can foster more reliable predictions, personalized diagnostics, and evidence-based management strategies.

## Methods

### Participants

Seventy children, adolescents, and adults with a genetic diagnosis of Phelan-McDermid syndrome were recruited at the Campus Bio-Medico University Hospital (Rome, Italy), and at the Interdepartmental Program “Autism 0–90” of the “G. Martino” University Hospital of Messina (Italy), where they were referred for medical follow-up and treatment. This sample underwent deep phenotyping and its clinical, demographic, and developmental characteristics have been recently described [46]. Five patients were excluded, because they carried a disruptive *SHANK3* point mutations, and two because they lacked exact deletion size determination by array-based technologies, although the presence of a deletion had been demonstrated by FISH. Ultimately, sixty-three patients were included in the present study, 31 females and 32 males, with mean (±S.D.) age 12.2 ± 10.1 years (range 1.7–44.0 years). Their demographic and genetic characteristics, intellectual level, and clinical DMS-5 diagnoses are summarized in Table 1. A formal DSM-5 diagnosis of ASD was given to 25.4% of cases, yielding an ASD prevalence that is lower than that reported in other studies [3, 47, 48] (see Discussion).

**Table 1:** Demographic information, genetic characteristics, intellectual level and clinical DMS-5 diagnosis of the sample (*N* = 63 unless otherwise specified)

Variable (sample size)			N	%
Sex	M		32	50.8%
	F		31	49.2%
Age	0–5 years		18	28.6%
	6–11 years		20	31.8%
	12–17 years		12	19.0%
	>18 years		13	20.6%
Chr 22q13.3 abnormalities	Simple deletions		55	87.3%
	Ring chr. with deletion		6	9.5%
	Unbalanced translocations		2	3.2%
Postzygotic mosaic deletions (≥ 30%)	Present		4	6.3%
	Absent		57	90.5%
DSM-5 clinical diagnoses:	Intellectual Disability		62	98.4%
	Motor Coordination Disorder/Dyspraxia		58	92.1%
	Autism Spectrum Disorder (ASD)		16	25.4%
	Bipolar Disorder	current	10	15.9%
		lifetime	12	19.0%
	ADHD		7	11.1%
	Oppositional Defiant Disorder		2	3.2%
	Depression		1	1.6%
	Generalized anxiety. panic disorder or simple phobia		1	1.6%
	Obsessive–Compulsive Disorder		1	1.6%
DSM-5 severity levels for ASD (*N* = 16)	Requiring support		3	18.8%
	Requiring substantial support		6	37.5%
	Requiring very substantial support		7	43.8%
ADOS categorization (*N* = 32)	Out of the spectrum		12	37.5%
	ASD spectrum NOT autism		5	15.6%
	Autism		15	46.9%
ADI-R B: verbal/non-verbal (*N* = 40)	Non-verbal		32	80.0%
	Verbal		8	20.0%

*DSM-5* Diagnostic and Statistical Manual of Mental Disorders −5, *ADHD* Attention Deficit/Hyperactivity Disorder, *ADOS* Autism Diagnostic Observation Schedule, *ADI-R* Autism Diagnostic Interview – Revised

This study was approved by the Institutional Review Board of University ‘‘Campus Bio-Medico’’ of Rome, Italy (protocol n. 14/98, first approval on April 28, 1998 and subsequent amendments) and by the Ethics Committee of Messina, Italy (protocol n. 22/17, approved on June 19, 2017). All parents gave written informed consent for themselves and for their affected offspring. All procedures performed in studies involving human participants are in accordance with the ethical standards of the institutional and/or national research committee and with the Helsinki declaration (2000).

### Assessment and outcome measures

All patients underwent an in-depth clinical assessment, as described [46]. Briefly, patient and family history were collected, and a thorough medical, neurological and neuropsychological evaluation was performed. Standardized instruments administered included Autism Diagnostic Observation Schedule – 2 (ADOS-2) [49] and Autism Diagnostic Interview – Revised (ADI-R) [50] for the evaluation of autistic symptomatology; the Repetitive Behavior Scale – Revised (RBS-R) [51] and the Short Sensory Profile (SSP) [52] for the evaluation of repetitive behaviors and sensory processing patterns, respectively; the Aberrant Behavior Checklist (ABC) [53] and the Child Behavior Checklist (CBCL) [54] to assess problematic behaviors; the Vineland Adaptive Behavior Scale-II (VABS-II), [55] to assess adaptive behaviors. Intellectual/developmental quotient was evaluated with the Griffiths Scales of Child Development- III [56], Wechsler Intelligence Scale for Children—Fourth Edition (WISC-IV) [57], or the Leiter-3 scale [58], depending on age and language skills. Finally, the Quality of Life in Autism Questionnaire (QOL-A) [59] and the World Health Organization's Quality of Life Questionnaire (WHOQOL) [60] were administered separately to mothers and fathers to assess parental quality of life.

### Genetic analysis

To determine deletion size, all 63 patients underwent CGH-array (Agilent 180 K or 400 K), as described in our previous studies [61, 62]. Karyotype analysis was performed to investigate the presence of a ring chromosome 22, which was detected in 6/63 (9.5%) deleted PMS patients (Table 1). The detection of postzygotic mosaic deletions by CGH-array displays variable sensitivity depending on array technology and deleted chromosomal region [63, 64]. In order to enhance reliability, we applied a minimum 30% mosaicism threshold [63], which identified 4/63 (6.5%) cases carrying postmitotic mosaic deletions (Table 1). All genomic coordinates correspond to the 2009 human genome build 19 (GRCh37/NCBI build 37.1). Deletion coordinates were plotted using the University of California at Santa Cruz Genome Browser (https://genome.ucsc.edu/). The length of all deletions is expressed as distance in kb from the telomere, identified as the hg19 genomic coordinate Chr22:51,244,566 (chr22_jh806586_fix).

### Genotype–phenotype correlations

**Fig. 1:**
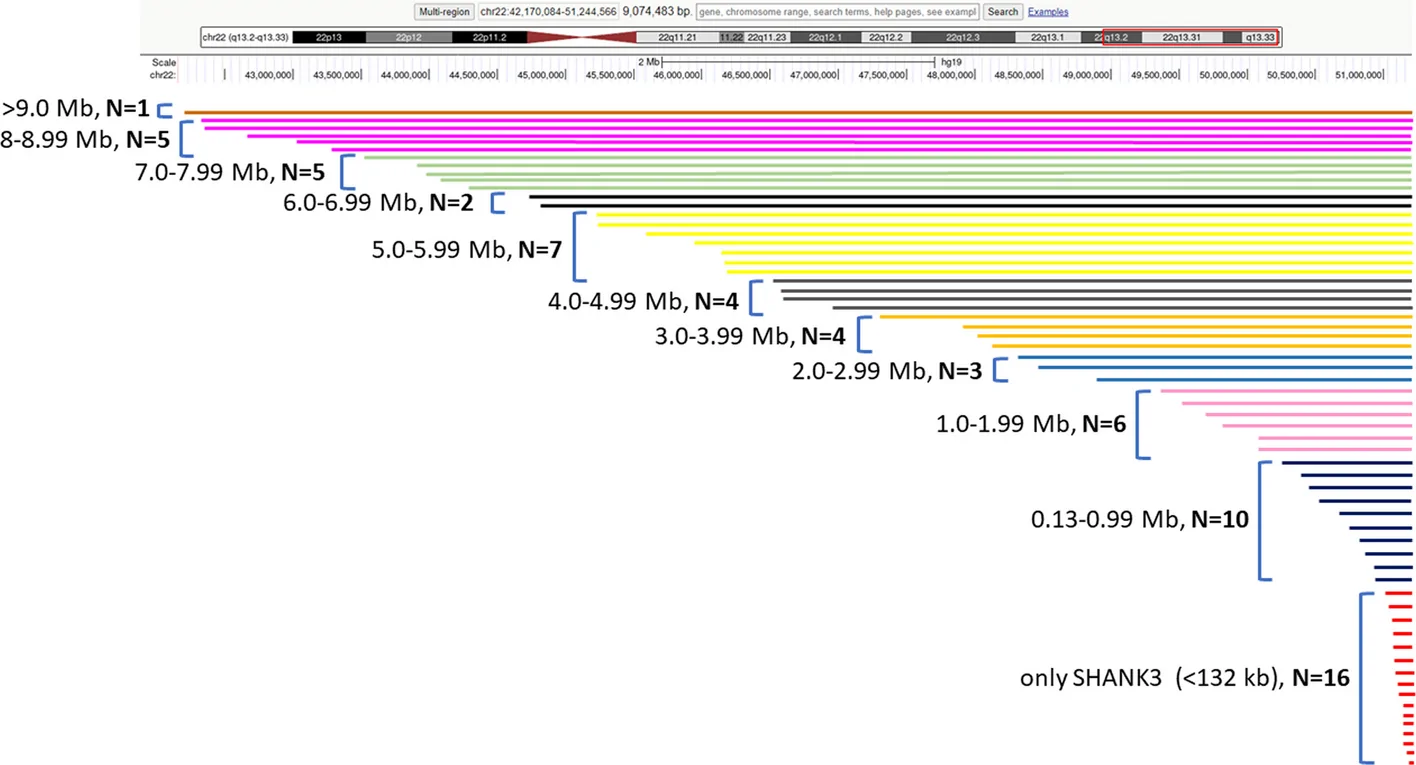
Size of terminal deletions at chromosome22q13 in our 63 PMS patients. Deletion size categories, as listed in Table 2, are displayed by different colors and the number of affected patients is indicated for each category. Genomic coordinates correspond to the hg19 genome assembly (Build 37)

Participants were distributed into eleven categories, according to deletion size (Fig. 1). The eleven categories were defined, as follows: patients carrying the smallest telomeric deletions, spanning only *SHANK3, ACR,* and *RABL2B*, fell into category n. 1 (< 132 kb in size, derived from nt 51,244,566–51,112,836/hg19). The remaining 10 categories include patients carrying increasingly larger deletions, with proximal breakpoint moving toward the centromere by 1 Mb per category, up to category 11, which includes the largest deletion found in our sample (9.008 Mb) (Fig. 1). Table 2 lists the most functionally relevant genes spanned by each chromosomal segment, as well as the number and percentages of participants carrying deletions which fall into each category. In simple terms, patients included in each category carry deletions spanning at least some telomeric genes present in that segment, plus all genes listed in the preceding categories (see Fig. 1 for graphical representation). Phenotypic variables significantly correlated with deletion size were not sought based on an a-priori hypothesis, but rather using an unbiased approach starting from 48 behavioral, medical, neurological, and patient history variables from our data set [46], after excluding results from tests and questionnaires. We first performed Kendall’s tau and an association χ^2^ test with the Monte Carlo procedure (10,000 iterations). Phenotypic variables reaching at least nominal significance with both tests were then further analyzed using two different statistical methods, in order to identify the associated candidate region, the maximum likelihood threshold, and the best candidate genes possibly responsible for each phenotype. For binary dichotomous phenotypic variables, the receiver operating characteristic (ROC) method [65] was used to evaluate all possible genomic breakpoint positions above which a given phenotype becomes aberrant. The maximum likelihood threshold was determined according to the Youden’s index (J = sensitivity + specificity—1), while the area under the curve (AUC) was calculated to determine whether the threshold identified was significantly capable to distinguish between “normal” and “abnormal” phenotype. The second method defined the best separation threshold as the optimal binary division that results in maximum heterogeneity between groups and homogeneity within groups. This method is similar to that of recursive partitioning, and the goodness of the division was measured by the complexity parameter (CP). For quantitative variables, the CP is equal to the proportion of variance explained by the binary partition, while for categorial variables it is proportional to the reduction of the generalized Gini impurity index. Statistical significance was set at *p* < 0.05, given the exploratory nature of this study and the available sample size. Nonetheless, all *p* < 0.001 also withstand a stringent Bonferroni correction (0.05/48 variables = 0.00104), which was also applied to the minimum p-value method. Statistical analyzes were performed in R, version 3.6.3 [66].

**Table 2:** Number (%) of patients by deletion size category (total *N* = 63). In each category, patients carry deletions spanning at least some telomeric genes present in that segment, plus all genes listed in the preceding categories (see Fig. 1 for graphical representation)

Deletion size	Category	Genes	Number of patients	%
< 132 kb	1	*RABL2B*	16	25.4%
		*SHANK3*		
132 kb – 0.99 Mb	2	*ARSA*	10	15.9%
		*MAPK8IP2*		
		*CHKB*		
		*CIMAP1B*		
		*TYMP*		
		*SCO2*		
		*SBF1*		
		*PLXNB2*		
		*TUBGCP6*		
		*MLC1*		
		*PIM3*		
		*ALG12*		
1–1.99 Mb	3	*BRD1*	6	9.5%
2–2.99 Mb	4	*TAFA5*	3	4.8%
3–3.99 Mb	5	*TBC1D22A*	4	6.3%
4–4.99 Mb	6	*CELSR1*	4	6.3%
		*TRMU*		
5–5.99 Mb	7	*ATXN10*	7	11.1%
		*FBLN1*		
		*UPK3A*		
6–6.99 Mb	8	*PRR5*	2	3.2%
		*PARVB*		
		*SAMM50*		
		*PNPLA3*		
7–7.99 Mb	9	*SULT4A1*	5	7.9%
		*MPPED1*		
		*SCUBE1*		
		*BIK*		
8–8.99 Mb	10	*A4GALT*	5	7.9%
		*CYB5R3*		
		*RNU12*		
		*TCF20*		
		*CYP2D6*		
		*NDUFA6*		
		*NAGA*		
		*TNFRSF13C*		
> 9 Mb	11	*SREBF2*	1	1.6%

### Prediction of the functional impact of haploinsufficiency for candidate genes

The functional impact of haploinsufficiency for the most promising candidates genes potentially involved in each phenotype was assessed using the “probability of being loss-of-function intolerant” (pLI) [67], the “Loss-of-function Observed/Expected Upper-bound Fraction” (LOEUF) score [68], and the “probability of haploinsufficiency” score (pHaplo) [69]. pLI and LOEUF were derived from gnomAD v4.1.0, pHaplo from UCSC Genome Browser hg38. In addition, transcriptional sensitivity to haploinsufficiency was empirically derived from the blood transcriptomic study by Breen et al., [70], who contrasted PMS patients with large vs small deletions.

## Results

Among the 63 patients included in this study, 55 (87.3%) carry simple terminal deletions of chromosome 22q13, six (9.5%) have a deletion in the context of a ring chromosome 22, and 2 carry unbalanced translocations (Table 1). Deletion size varied from 25 kb to 9.008 Mb (median: 1.847 kb) [46].

### Phenotypic variables associated with deletion size

Fourteen phenotypic variables, including some well-known PMS characteristics and several clinically relevant non-specific features, displayed a significant association with deletion size applying *both* Kendall’s tau and an association χ^2^ test with Monte Carlo exact p-value estimation (Table 3). For the majority of these variables, both nominal *p*-values were < 0.001 (< 0.05 after Bonferroni’s correction). The fourteen variables associated with deletion size and listed in Table 3 fall into five domains:

- language development:
  presence/absence of verbal language;
  level of verbal language (no verbal language/words only/words and sentences);
  first sentences acquisition timing.
- motor function and development:
  timing of motor development (typical/delayed/not walking);
  gait (normal/abnormal/not walking);
  muscle strength (normal/reduced).
- social cognition;
  eye contact (normal/inconsistent/absent);
  exchange gesture (complete/incomplete/absent);
  joint attention (complete/incomplete/absent).
- medical issues:
  structural abnormalities on brain MRI;
  presence/absence of an infectious disease at the onset of behavioral symptoms;
  renal abnormalities and malformations:
  dysmorphisms.
- comorbid disorders;
  lifetime diagnosis of bipolar disorder (present/absent).

**Table 3:** Phenotypic variables significantly associated with deletion size by both Kendall’s tau and association exact χ^2 ^test. Chromosomal positions, expressed as distance in kb from the telomere (hg19 genomic coordinate Chr22:51,244,566), hosting maximum likelihood candidate genes for each phenotype were determined using the minimum *p*-value and ROC curve methods. Maximum likelihood thresholds are highlighted in bold.

Variable (N)	Kendall’s tau (exact nominal p)	Association χ^2^ test (exact nominal p)	Minimum P-value method			ROC curve	
			Best cut-off	Region of association	Minimum P (with Bonferroni)	Peak	AUC
Presence/absence of verbal language (63)	−0.390 (*p* < 0.001)	9.929 (*p* < 0.001)	**114 kb**	68–5,614 kb	3.0 × 10^–5^ (0.00135)	**1,059 kb**	0.8183
Verbal language level (sentences/words only/non verbal) (63)	−0.407 (*p* < 0.001)	11.150 (*p* < 0.001)	**114 kb**	68–5,013 kb	1.9 × 10^–6^ (9.9 × 10^–5^)	NA	NA
First sentences acquisition timing (63)	0.483 (*p* < 0.001)	15.914 (*p* < 0.001)	**114 kb**	67–4,743 kb	1.1 × 10^–7^ (6.0 × 10^–6^)	NA	NA
Motor development timing (63)	0.632 (*p* < 0.001)	32.962 (*p* < 0.001)	**3,039 kb**	67–6,529 kb	1.0 × 10^–5^ (0.00028)	**3,039 kb**	0.8597
Gait (normal/abnormal/absent) (55)	0.516 (*p* < 0.001)	20.585 (*p* < 0.001)	**5,065 kb**	106–8,527 kb	< 1.0 × 10^–5^ (0.00011)	NA	NA
Muscle strength (49)	0.256 (*p* = 0.038)	7.230 (*p* = 0.005)	**7,363 kb**	2,953–8,234 kb	1.0 × 10^–4^ (0.0041)	**5,013 kb**	0.7963
Eye contact (53)	0.312 (*p* = 0.006)	9.004 (*p* = 0.001)	**3,282 kb**	2,953–5,013 kb	6.9 × 10^–3^ (0.29595)	**3,282 kb**	0.6904
Reciprocal object exchange gesture (47)	0.420 (*P* < 0.001)	12.865 (*p* < 0.001)	**3,282 kb**	106–6,529 kb	1.7 × 10^–4^ (0.00658)	**3,282 kb**	0.7329
Joint attention (49)	0.272 (*p* = 0.023)	6.367 (*p* = 0.004)	**4,743 kb**	3,039–5,912 kb	3.7 × 10^–3^ (0.14633)	**3,282 kb**	0.7055
Presence/absence of infectious pathology at behavioral symptom onset (63)	0.346 (*p* = 0.002)	7.586 (*p* = 0.003)	**253 kb**	83–4,357 kb	3.7 × 10^–4^ (0.01970)	**1,059 kb**	0.7161
Brain structural abnormalities on MRI (60)	0.368 (*p* < 0.001)	14.556 (*p* < 0.001)	**451 kb** **5,024 kb**	68–7,205 kb 68–7,755 kb	4.0 × 10^–5^ (0.00140) 1.0 × 10^–5^ (0.00065)	**833 kb**	0.9167
Renal and urinary tract anomalies (58)	0.325 (*p* = 0.003)	9.317 (*p* = 0.002)	**4,743 kb**	127–8,013 kb	4.2 × 10^–3^ (0.19367)	**2,953 kb**	0.7457
Dysmorphisms (major/minor/absent) (53)	0.417 (*p* < 0.001)	16.298 (*p* < 0.001)	**1,860 kb**	451–6,529 kb	4.6 × 10^–6^ (0.0002)	**NA**	NA
Comorbid lifelong Bipolar Disorder (62)	−0.234 (*p* = 0.034)	2.908 (*p* = 0.044)	**67 kb**	56–1,544 kb	1.2 × 10^–3^ (0.06067)	**114 kb**	0.7437

### Genotype–phenotype correlation: identification of candidate genes

ROC curve analysis and the optimal separation method led to the identification of one to three “best cut-offs” or “ROC curve peaks” along chromosome 22q13 for each variable (Table 3): for uniformity, these peaks will hereby be defined “maximum likelihood thresholds”. In simple terms, the transition from normal to altered phenotype provides relevant information on candidate genes that, if haploinsufficient, significantly enhance the probability of appearance of the phenotype under scrutiny.

**Fig. 2:**
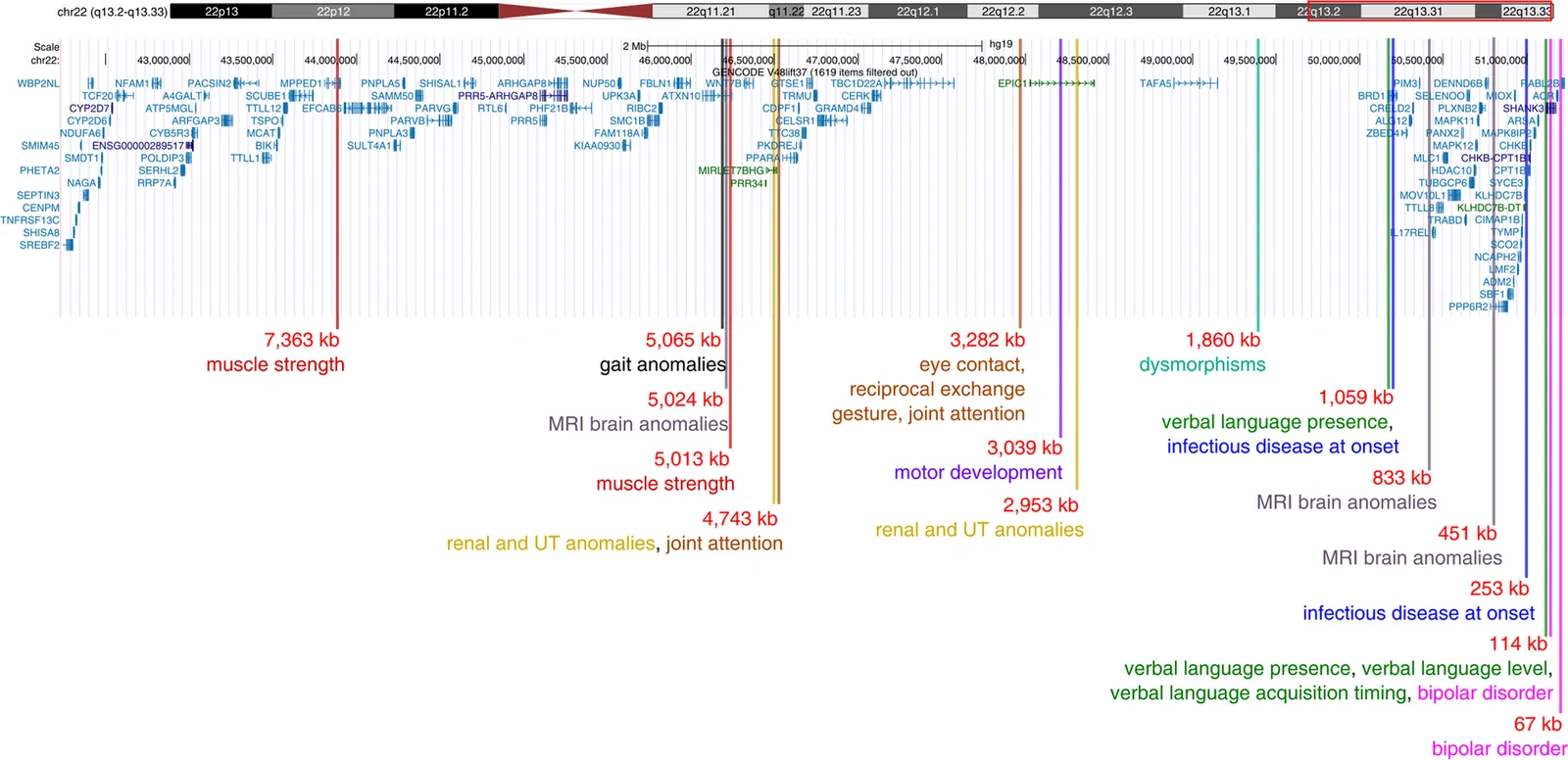
Position of the maximum likelihood thresholds in chromosome 22q13 for all phenotypic variables significantly associated with deletion size, distinguished by color code (see text)

Maximum likelihood thresholds are displayed in Fig. 2. Patient counts by deletion size category are presented in Tables 4, 5, 6, 7, 8, 9, where the transition from normal to altered phenotype is emphasized in bold and thick lines highlight the boundary closest to each maximum likelihood threshold (i.e., best cut-offs or ROC curve peaks, as listed in Table 3 and displayed in Fig. 2). Detailed close-up images of each maximum likelihood threshold are provided in Suppl. Figures S1-S9.

**Table 4:**
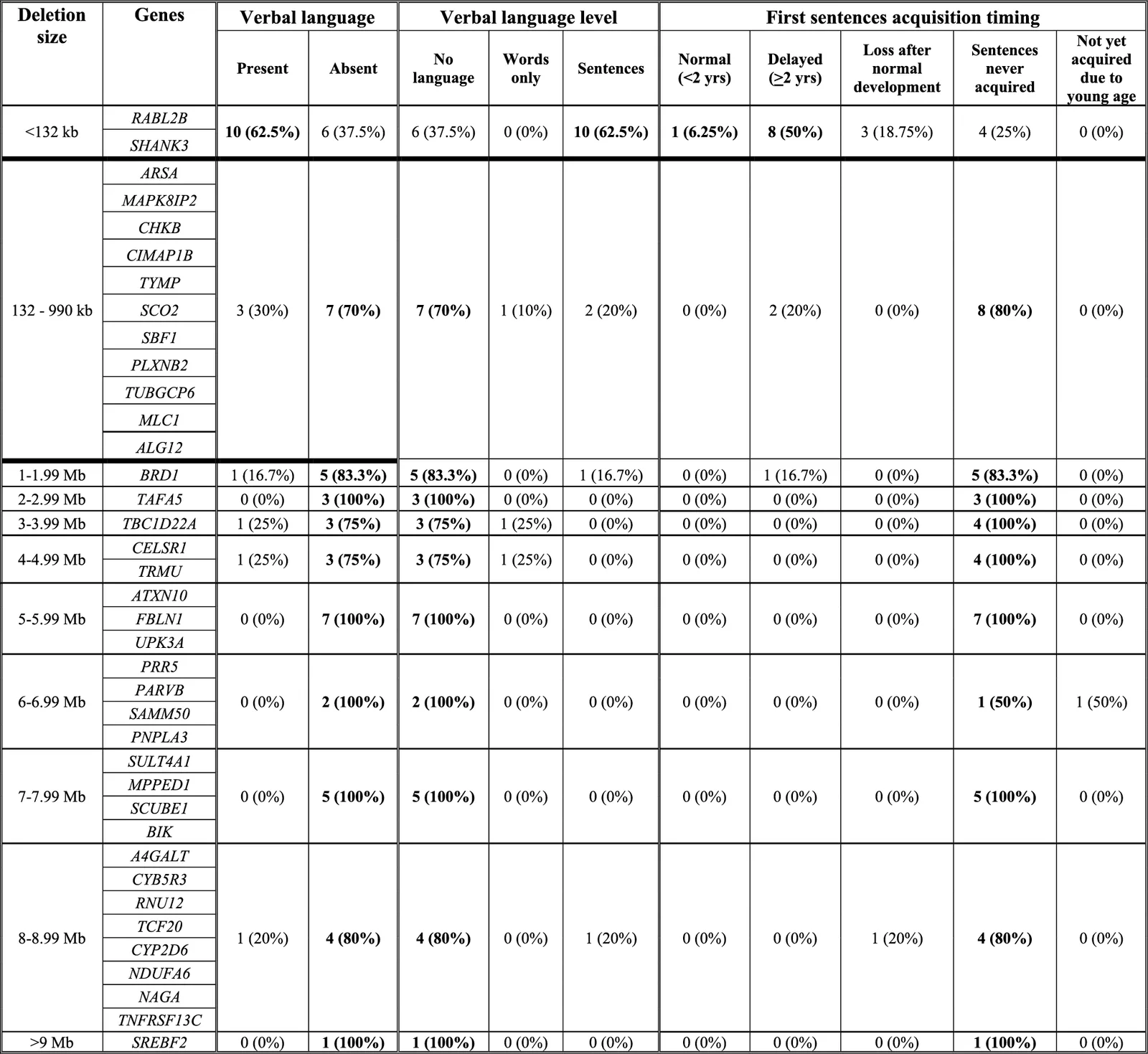
*Language development: *distribution of “verbal language present/absent”, “verbal language level” (non-verbal vs words only vs produces sentences), and “timing of acquisition of first sentences” by deletion size category (total *N*=63). For each variable, thick lines delineate the boundary closest to the maximum likelihood thresholds (i.e., best cut-offs or ROC curve peaks) listed in Table 3 and displayed in Fig. 2, while data highlighted in bold emphasize the transition from normal to altered phenotype

#### Language development

All variables concerning the language domain, including (a) presence/absence of speech, (b) level of verbal language development (non-verbal/words only/sentences), and (c) timing of first sentences acquisition, shared one maximum likelihood threshold located at 114 kb from the telomere (Tables 3 and 4). This threshold is located within the *SHANK3* gene (Fig. 2, dark green; Suppl. Fig. S1A for a detailed view). This intragenic peak, paired with the presence of nonverbal patients in all categories, including category 1 (Table 4), indicates that *SHANK3* plays a major role in verbal language impairment. In addition, ROC curves for “presence/absence of speech “ also identify a second peak at 1,059 kb from the telomere (Tables 3 and 4), falling within the *BRD1* gene (Fig. 2, dark green; Suppl. Fig. S1B).

#### Motor function and development

Motor development timing was normal in approximately 40% of our PMS patients and delayed in 50%, whereas autonomous walking has never been acquired by 10% [46]. The timing of motor development is strongly correlated with deletion size (*p* < 0.001, Table 3), which alone explains 47.6% of the variance: 24/35 (68.6%) patients with deletions < 3 Mb had normal motor development timing, compared to only 1/28 (3.6%) carrying deletions ≥ 3 Mb (Table 5). The maximum likelihood threshold is located in a gene-poor region at 3,039 kb from the telomere (*p* = 2.8 × 10^–4^ after Bonferroni’s correction; AUC = 0.86) (Table 3), within the oncogenic lncRNA *EPIC1* and roughly 700 kb away from *TBC1D22A*, the first upstream coding gene (Fig. 2; Suppl. Fig. S2).

**Table 5:**
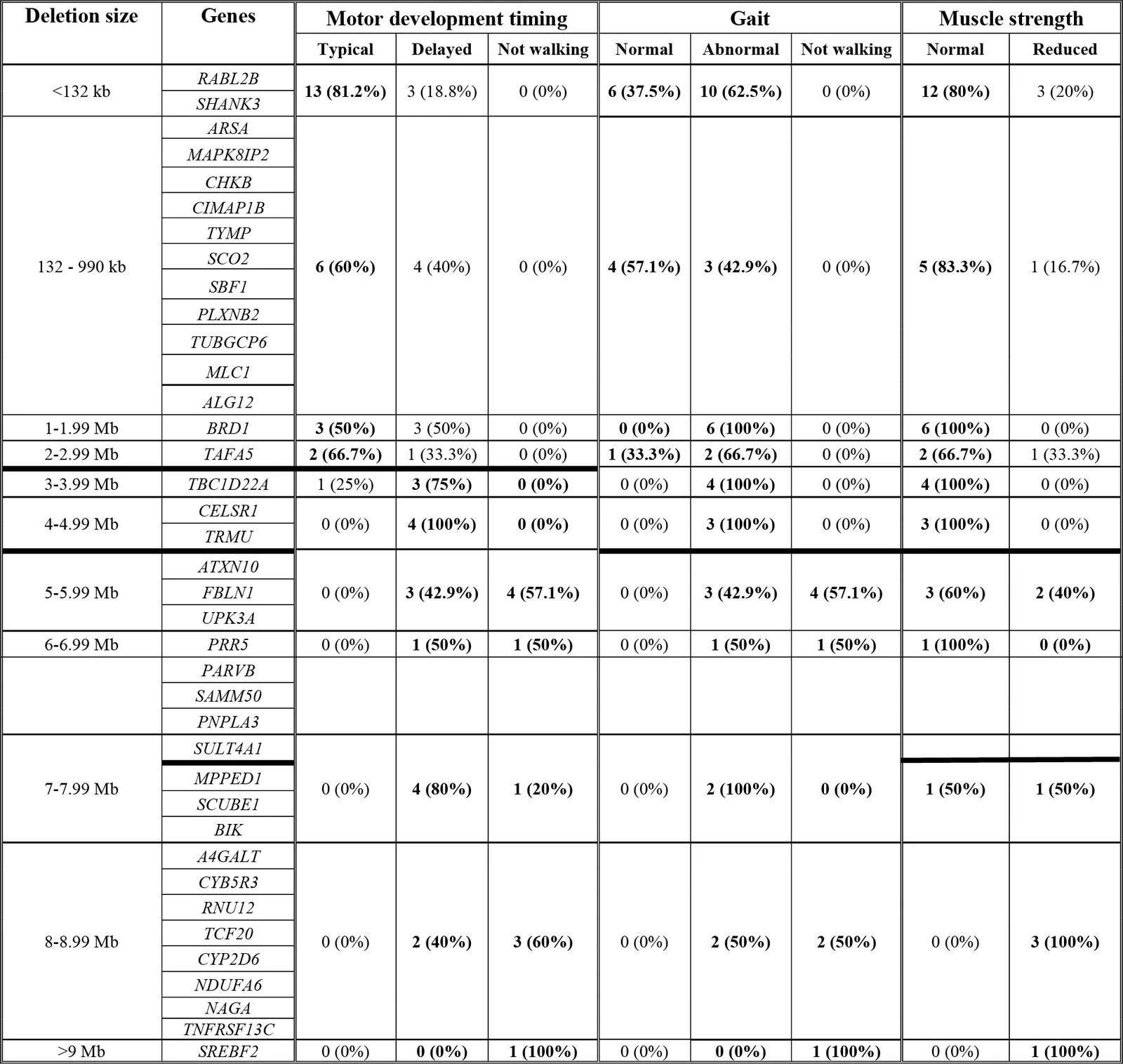
*Motor function and development: *distribution of motor development timing (*N*=63 patients), gait (*N*=55), and muscle strength (*N*=49, assessed by neurological examination, by deletion size. For each variable, thick lines delineate the boundary closest to the maximum likelihood thresholds (i.e., best cut-offs or ROC curve peaks) listed in Table 3 and displayed in Fig. 2, while data highlighted in bold emphasize the transition of prevalence from normal to altered phenotype

Both gait and muscle strength, assessed clinically during the neurological examination (see Suppl. Table 4 in ref. 46], are significantly correlated with deletion size and display maximum likelihood thresholds with practically superimposable locations, i.e. 5,065 kb and 5,013 kb from the telomere, respectively (Tables 3 and 5). The influence of this chromosomal region is especially evident for gait, where all 39 (100%) patients with a deletion size < 5 Mb can walk independently, albeit often with abnormal gait (28/39, 71.8%) while 8/16 (50%) with larger deletions cannot walk independently at all and the remaining 8/16 (50%) display an abnormal gait (Table 5). Within or just centromeric to this threshold are located two very important neuronal genes, *ATXN10* and *FBLN1* (Fig. 2 and Suppl. Fig. S3A). For muscle strength only, a second threshold located at 7,363 kb was identified (Tables 3 and 5), falling within the *MPPED1* gene and 140 kb downstream of *SCUBE1* (Suppl. Fig. S3B).

#### Social cognition (eye contact, exchange gesture, joint attention)

Social cognition deficits, involving eye contact, joint attention, theory of mind, and non-verbal communication, are frequently observed in PMS, especially when a comorbid ASD is diagnosed [3, 4, 10, 18, 29, 33, 34, 46–48]. Deletion size was significantly correlated with deficits in eye contact, reciprocal object exchange gesture (which requires theory of mind), and joint attention (Table 3). All three variables displayed a more benign course in patients with deletions smaller than 3 Mb (Table 6). The maximum likelihood threshold consistently shared by all three variables is located at 3,282 kb from the telomere (Table 3). The strongest candidate gene centromeric to this threshold is *TBC1D22A* (Fig. 2), described above also in reference to motor development timing (Fig. 2, Suppl. Fig. S4). Only for joint attention, a second significant threshold was found at 4,743 kb (Table 2, Fig. 2). This second threshold is shared with renal anomalies, suggesting possible contributions to the autism phenotype by the long noncoding RNAs (PRR34, MIRLET7BHG) and antisense RNAs discussed below in reference to renal malformations, or by upstream coding genes, such as *WNT7B*, *ATXN10*, and/or *FBLN1*, the latter two also implicated in gait and muscle strength deficits (Suppl. Fig. S3A).

**Table 6:**
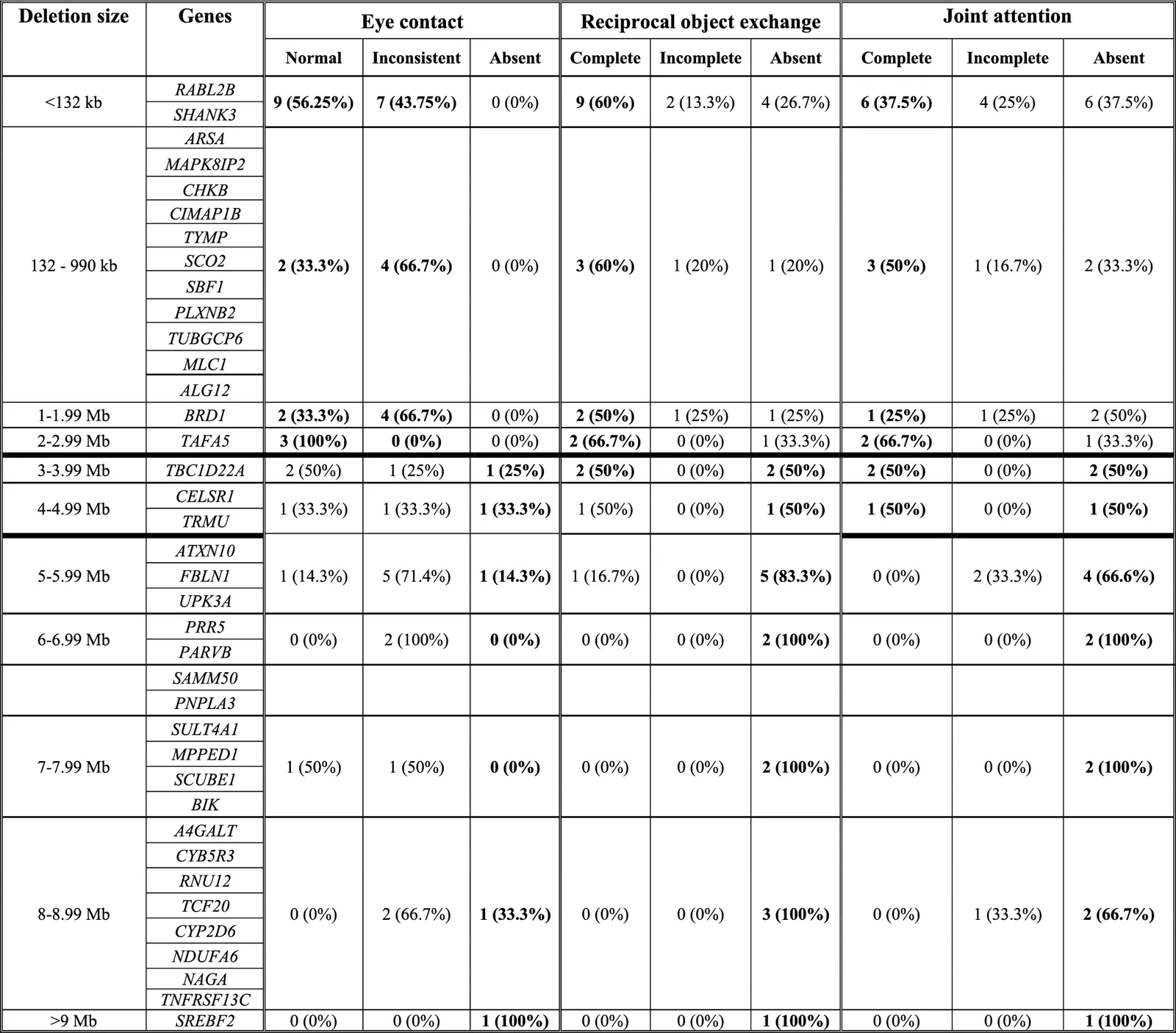
*Social cognition*: distribution of eye contact (total *N*=53). reciprocal object exchange gesture (total *N*=47), and joint attention (total *N*=49) by deletion size. For each variable, thick lines delineate the boundary closest to the maximum likelihood thresholds (i.e., best cut-offs or ROC curve peaks) listed in Table 3 and displayed in Fig. 2, while data highlighted in bold emphasize the transition from normal to altered phenotype

#### Medical issues

##### Structural abnormalities on brain MRI

The presence of single or multiple structural brain abnormalities on MRI was strongly associated with deletion size (*p* < 0.001, Table 3). A detailed distribution of specific structural abnormalities by deletion size category is provided in Suppl. Table 1. The minimum P-value method and/or ROC curves identified three maximum likelihood thresholds, located at 451 kb, 833 kb, and 5,024 kb, respectively (Fig. 2). The importance of these three thresholds is clear when considering patient distributions in Table 7: multiple brain structural anomalies are present in 6/24 (25.0%) patients carrying deletions < 1 Mb (categories 1 and 2), 6/16 (37.5%) with deletion size 1–4.99 Mb (categories 3–6), and 18/20 (90.0%) with deletions ≥ 5 Mb (categories 7–11) (Table 7, see Suppl. Table 1). Similarly, 8/12 (66.7%) cases with an MRI devoid of any evident structural abnormality carry very small deletions, i.e. < 132 kb (Table 7, Suppl. Table 1).

**Table 7:**
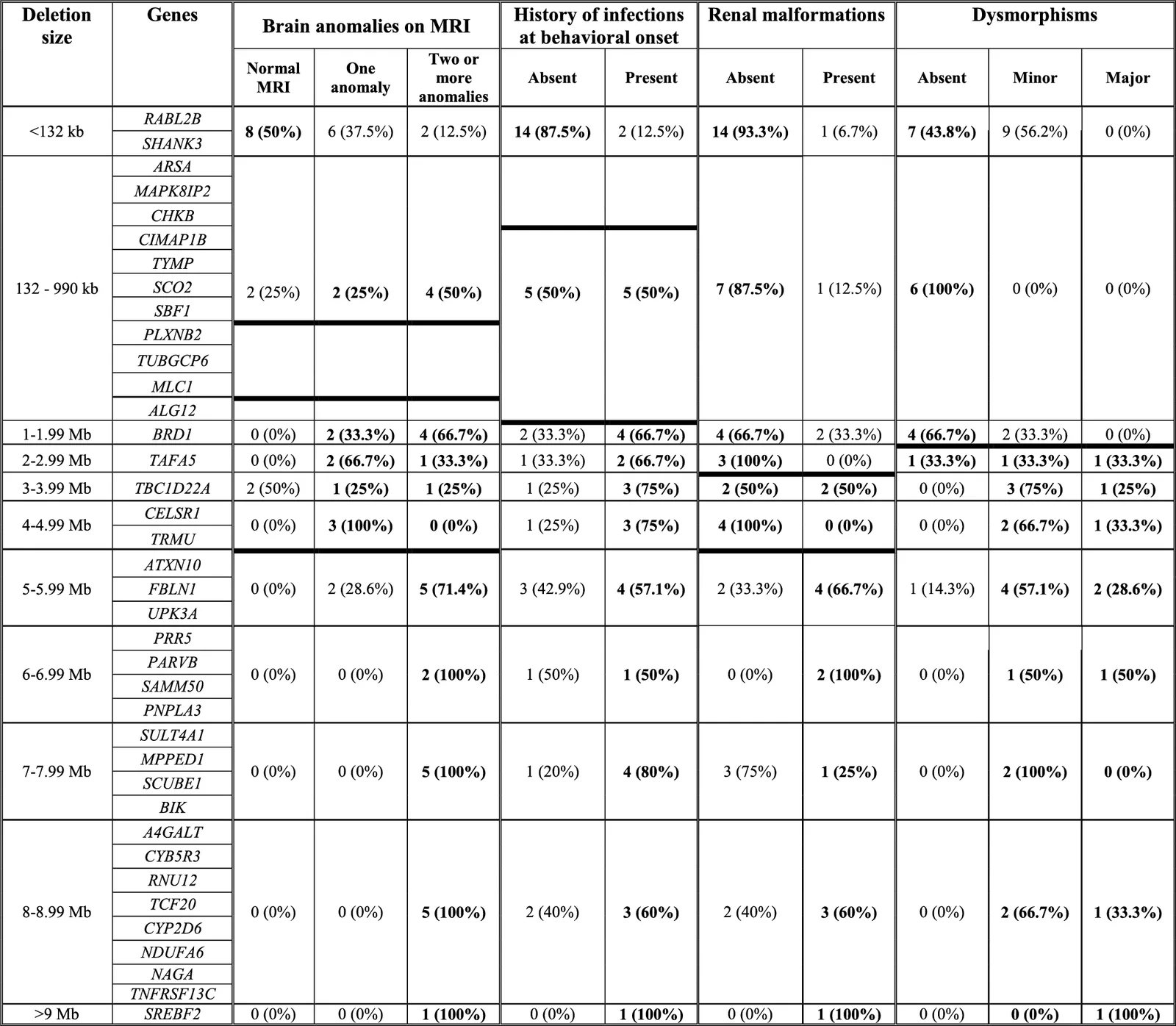
*Medical issues:* distribution of brain structural abnormalities on MRI (*N* = 60), presence/absence of infectious pathology coincident with the onset of behavioral manifestations (*N* = 63), renal and upper urinary malformations (*N* = 58), and dysmorphisms (*N* = 53) by deletion size category. Data highlighted in bold emphasize the transition of prevalence from normal to altered phenotype

The first threshold falls within the *PPP6R2* gene, involved in chromosomal stability during the mitotic cycle (Fig. 2, Suppl. Fig. S5A). Centromeric to this gene, at only 66.8 and 86.3 kb from *PPP6R2*, are located *DENND6B*, which encodes for a guanine nucleotide exchange factor (GEF) for RAB14, and most importantly the morphogenetic *PLXNB2*, involved in embryonic brain and kidney development (see Discussion) (Suppl. Fig. S5A). The second threshold falls at 53.6 and 99.2 kb telomeric from *PIM3* and *ALG12*, respectively, while *BRD1* is located further upstream at 192.8 kb (Fig. 2, Suppl. Fig. S5B). *PIM3* belong to the Ser/Thr protein kinase family and PIM subfamily, involved in cell proliferation and survival; *ALG12* belongs to the glycosyltransferase 22 family and is involved in protein glycosylation also in the central nervous system (CNS). Available evidence does not support probable roles for other genes located in this chromosomal segment (see Discussion). The third peak is located at 5,024 kb and falls within the *ATXN10* sequence, as occurs with the thresholds for muscle strength (5,013 kb), gait (5,065 kb), and joint attention (4,743 kb) (Fig. 2, Suppl. Fig. S5C).

##### Infectious disease coincident with the onset of behavioral symptoms

PMS can become apparent already in neonates, when prominent muscle hypotonia and developmental delay are present [1, 2]. However, for approximately half of our sample, motor and behavioral development did not immediately raise concern both in parents and in the pediatrician, until anomalies emerged later on, often during or immediately after an infectious episode, especially ear-nose-throat or lower airway infections [46]. Deletion size is significantly correlated with the latter course (Table 3)*.* In fact, a first observation of clear neurobehavioral manifestations co-occurring with an infectious episode was rarely reported for the vast majority of patients with small deletions affecting only *SHANK3* (*N* = 2/16, 12.5%), but became significantly more frequent among carriers of larger deletions (Table 7). Statistical analysis identified two maximum likelihood thresholds (Table 3, Fig. 2): the first threshold is located at 253 kb, spanning the locus encompassing *KLHDC7B* and its lncRNA *KLHDC7B-DT*, and only 20 kb telomeric from *CIMAP1B,* previously also called *ODF3B* (Suppl. Fig. S6). The second peak is located at 1,059 kb, falls within *BRD1*, and coincides with the second maximum likelihood threshold described above for presence/absence of verbal language (Fig. 2, Suppl. Figure 1B). Interestingly, *KLHDC7B*, *KLHDC7B-DT*, *CIMAP1B* and *BRD1* all play important roles in immune function (see Discussion).

##### Renal and upper urinary malformations

Malformations of the kidney or upper urinary tract, assessed by abdominal ultrasound, were found in approximately one third of our sample, as previously reported [46]. This phenotypic feature was significantly associated with deletion size (Table 3). Our results show that renal and upper urinary malformations are present in a small minority (4/32, 12.5%) of patients carrying deletions < 3 Mb in size, increase among patients with deletions of 3–5 Mb (2/8, 25%), and become common among carriers of deletions greater than 5 Mb (11/18, 61.1%) (Table 7). Four patients (6.9%) found to carry kidney stones in the absence of upper urinary tract malformations were included in Table 7, two in category 1 (deletion size < 132 kb) and two in category 5 (3–3.99 Mb). Two significant deletion thresholds were found for renal abnormalities, the first located at 2,953 kb, and the second at 4,743 kb (Table 3, Fig. 2, Suppl. Figures 7 A and 7B). The first threshold is located in a relatively gene-poor region and falls into the distal sequence of the longer isoforms of the oncogenic lncRNA *EPIC1* (Fig. 2, Suppl. Figure 7 A). It is only 86 kb telomeric from the maximum likelihood threshold strongly associated with motor development timing (Fig. 2, Suppl. Fig. S2). Also the second threshold falls into a chromosomal region encompassing long noncoding RNAs (PRR34, MIRLET7BHG) and a cluster of antisense RNA (Fig. 2, Suppl. Figure 7B). Further downstream, at 128.6 kb, is located the *WNT7B* gene, which is highly expressed in the urinary tract and is involved in patterning during embryogenesis (see Discussion).

##### Dysmorphisms

We detected a very significant correlation between deletion size and the presence of dysmorphisms (*p* < 0.001, Table 3). Deletions smaller than 2 Mb were characterized by 17/28 (60.7%) cases displaying no noticeable dysmorphism and by the complete absence of major dysmorphisms (Table 7). Instead, starting from the 2.0–2.9 Mb category onward, the opposite trend was observed, namely only 2/25 (8.0%) cases display no dysmorphism and 8/25 (32.0%) display major dysmorphisms (Table 7). Statistical analyses detect a single maximum likelihood threshold at 1,860 kb (Fig. 2, Suppl. Fig. S8), beyond which deletions are associated with an increased frequency and severity of dysmorphisms. The first coding gene beyond this threshold is *TAFA5*, located at 236 kb (Suppl. Fig. S8).

#### Psychiatric comorbidities: lifetime diagnosis of bipolar disorder

PMS is associated with an increased prevalence of bipolar disorder, which is a frequent cause of regression in adolescents and young adults [22–25]. Also in our sample, as many as 12/63 (19.0%) PMS patients have received a lifetime diagnosis of bipolar disorder (Table 1). A small deletion involving only *RABL2B*, *ACR* and variable segments of *SHANK3* is present in the majority (7/12, 58.3%) of these PMS patients, while only 5/12 (41.7%) carry larger deletions (Table 8). Furthermore, among the 16 patients carrying small deletions (i.e., < 110 kb), those with a comorbid diagnosis of bipolar disorder represent a sizable minority (7/16 = 43.8%) (Table 8). Not surprisingly, both the 67 kb and the 114 kb maximum likelihood thresholds are extremely close or fall within the *SHANK3* gene (Fig. 2, Suppl. Fig. S9), similarly to the threshold already presented for language development (Fig. 2, Suppl. Figure 1A). Finally, this phenotype displays the smallest associated region among our fourteen variables, spanning from 43 kb to 1.531 Mb (Table 3). Hence collectively also for bipolar disorder, *SHANK3* appears to play a major role, possibly without requiring contributions by other genes.

**Table 8:**
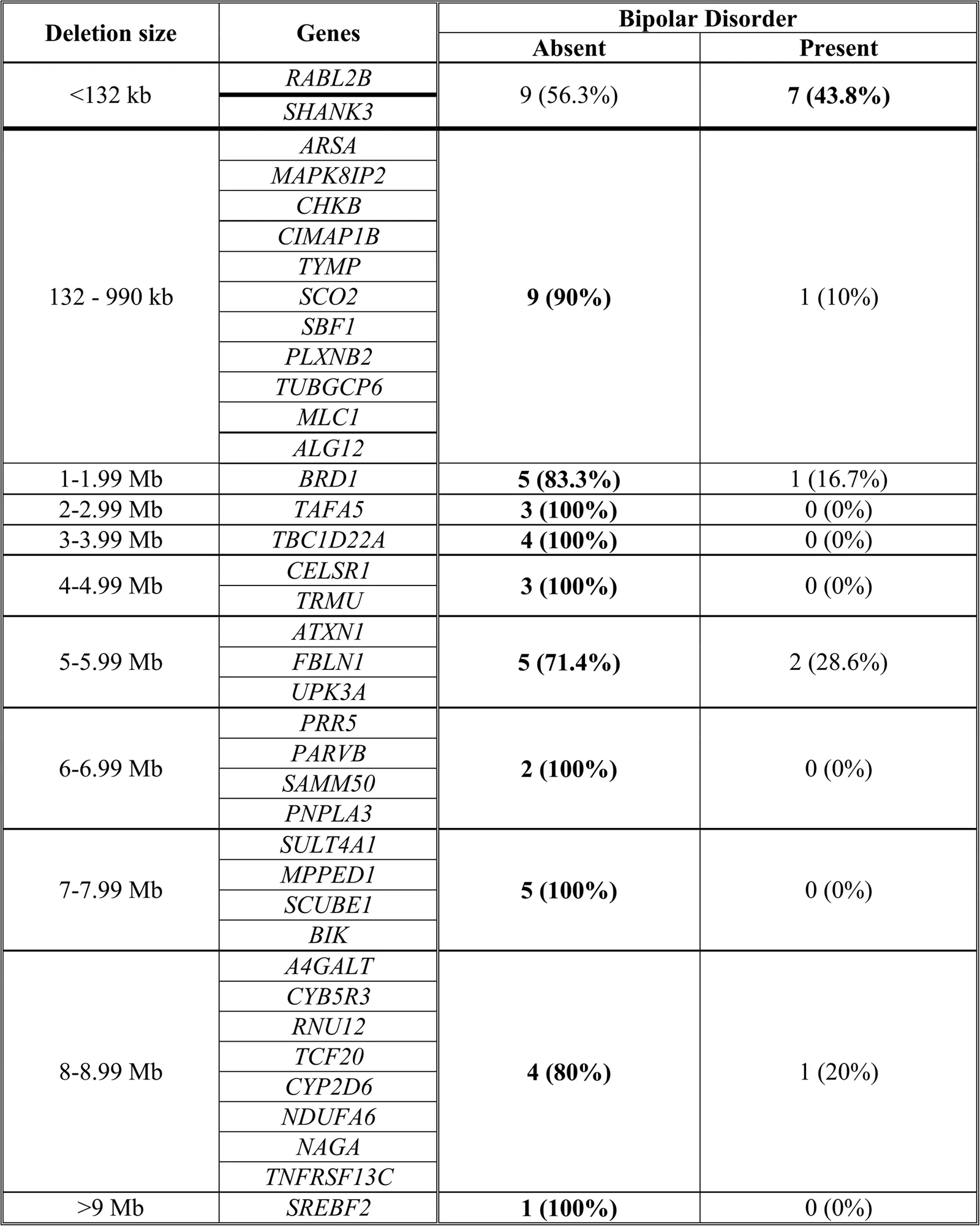
Psychiatric comorbidities: distribution of presence/absence of a lifetime diagnosis of bipolar disorder by deletion size category (total *N* = 62). Data highlighted in bold emphasize the transition of prevalence from normal to altered phenotype

### Prediction of functional impact for haploinsufficiency at the proposed candidate genes

The functional impact of haploinsufficiency for the most promising candidates genes was assessed using computational damage prediction scores, namely pLI [67], LOEUF [68], and pHaplo [69], in addition to integrating also empirical data on transcriptional sensitivity to haploinsufficiency from the blood transcriptomic study by Breen et al., [70] (Table 9). Most candidate genes have a low pLI, with the only exception of *BRD1* and *FBLN1*, displaying a pLI > 0.9 compatible with intolerance to loss-of-function by this metric (Table 9). Importantly, the pLI of the established disease modifier *CELSR1*, associated with lymphedema in PMS [13], is 0 according to gnomAD 4.1.0 (Table 9), confirming the frequent occurance of false-negatives with this metric [71]. In addition to *BRD1* and *FBLN1,* LOEUF also detected the well-known morphogenetic gene *PLXNB2* as a constrained gene, and confirmed *CELSR1* (Table 9). Instead, pHaplo and the blood transcriptomic data from Breen et al. [70], two parameters better fit to predict gene dosage sensitivity for pathomorphic non-pathogenic genes spanning CNVs, indicated that all the candidate genes proposed in the present study either have pHaplo ≥ 0.86 or are dosage-sensitive at the transcriptional level, except for *DENND6B*, *UPK3A*, and *MPPED1,* which are not expressed in blood and for which we thus have no information (Table 9). This analysis lends support to gene dosage sensitivity for the candidate genes most compatible with our maximum likelihood threshold, especially if they are considered in the context of a large deletion (pHaplo), or if assessed at the transcriptional level [70].

**Table 9:** Impact of haploinsufficiency for the candidate pathomorphic genes proposed in this study and for two genes with known effects (*CELSR1* and *TCF20*), as predicted by pLI [67] and LOEUF [68] from gnomAD v4.1.0, pHaplo [69] from UCSC genome browser hg38, and by blood transcriptomic analysis carried out by Breen et al. [70]. Values indicating genetic constraint according to each metric or reduced gene expression in blood are highlighted in bold

Candidate genes	pLI cutoff: ≥ 0.9	LOEUF cutoff: ≤ 0.6	pHaplo cutoff: ≥ 0.86	Expressed in blood (Breen et al. 2023)	Downregulated in large chromosome 22q13 deletions (Breen et al. 2023)
*KLHDC7B*	0.02	1.95	0.6	**Y**	**Y**
*CIMAP1B*	0	1.52	**0.97**	**Y**	**Y**
*PPP6R2*	0	0.82	**0.88**	**Y**	**Y**
*DENND6B*	0	1.21	0.57	N	/
*PLXNB2*	0	**0.55**	**0.94**	**Y**	**Y**
*PIM3*	0	1	**0.93**	**Y**	**Y**
*ALG12*	0	0.96	0.58	**Y**	**Y**
*BRD1*	**1**	**0.34**	**0.99**	**Y**	**Y**
*TAFA5*	0.48	0.82	**0.89**	N	/
*TBC1D22A*	0	0.88	**0.96**	**Y**	**Y**
*WNT7B*	0	0.76	**0.93**	N	/
*ATXN10*	0	0.72	0.82	**Y**	**Y**
*FBLN1*	**0.95**	**0.5**	**0.98**	N	/
*UPK3A*	0	1.57	0.38	N	/
*MPPED1*	0.05	0.7	0.72	N	/
*SCUBE1*	0	0.65	**0.97**	N	/

Known genes	pLI cutoff: ≥ 0.9	LOEUF cutoff: ≤ 0.6	pHaplo cutoff: ≥ 0.86	Expressed in blood (Breen et al. 2023)	Downregulated in large chromosome 22q13 deletions (Breen et al. 2023)
*CELSR1*	0	**0.56**	**0.99**	**Y**	**N**
*TCF20*	**1**	**0.05**	**0.97**	N	/

*Abbreviations*: Y = yes, N = no,/= not assessed

## Discussion

The ultimate purpose of this study is to explore the heuristic potential of genetic information provided by array-based technologies, in fostering more reliable prognostic predictions and better clinical management of PMS patients carrying chromosome 22q13 deletions. This effort follows our long-standing interest toward expanding the role of genetics beyond the narrow boundaries of the diagnostic process of neurodevelopmental disorders, into “actionable genomics” in clinical practice [72]. To this aim, here we first define a set of clinical variables significantly associated with chromosome 22q13 terminal deletion size, and then we analyze their genotype–phenotype correlation. Our results support the role of several strong candidate genes in shaping the clinical presentation and developmental trajectory of individuals with PMS, in conjunction with and in addition to *SHANK3*. These candidate genes seemingly influence several clinically-relevant functional domains.

### Speech and language delay

Speech impairment and/or language delay represent a major clinical characteristic of PMS, reported in 50% to 80% of the patients [43, 73], and affecting the vast majority of our sample (Tables 1 and 4). The great interindividual variability in verbal language skills among patients with PMS has made it difficult to identify a specific deleted gene or regions critical for this function [74]. Initially, case reports of a boy and a woman carrying a translocation disrupting *SHANK3* both described different aspects of speech delay [9, 43]. In addition, individuals with small deletions were reported to display greater phenotypic variability in language development, ranging from lack of verbal language to use of sentences and less hampered communication skills [73–76]. Deletions greater than 5–6 Mb have been associated with complete lack of verbal language [77]. Our study replicates and extends these observations, identifying at least two critical regions for spoken language in chromosome 22q13. On the one hand, small deletions with breakpoints within *SHANK3* are found in patients widely ranging from non-verbal (6/16, 37.5%) to speaking full sentences (10/16, 62.5%). On the other hand, almost all subjects (33/37, 89.2%) carrying deletions > 1 Mb from the telomeric end of chromosome 22 are nonverbal (Table 4).

The first threshold (Fig. 2, Suppl. Figure 1A) confirms the importance of *SHANK3* (OMIM*606,230) in speech abnormalities associated with PMS [9, 35, 43, 78, 79]. Imaging studies using DTI have documented altered myelination in the inferior frontal occipital fasciculus and in the uncinate fasciculus [80], the former critical for semantic processing, reading, and basic naming [81], and the latter for language comprehension, long-term memory, and general speech performance [73]. In mice, reduced expression of *SHANK3* was correlated with similar myelination abnormalities [80]. Also *SHANK3* indels mutant macaques exhibit reduced vocalizations, in addition to repetitive behaviors and abnormal social interactions [82]. Hence, this first threshold is not surprising, given pivotal role of *SHANK3* in neurodevelopment, synaptogenesis, and synaptic management [83].

On the other hand, our sample distributions (Table 4) and the second maximum likelihood threshold for verbal language impairment located at 1,059 kb, falling within the candidate gene *BRD1* (Fig. 2, Suppl. Figure 1B)*,* demonstrate that deletions greater than 1 Mb in size are already almost invariably associated with absent verbal language, previously linked to larger deletions exceeding 5 Mb [74, 75, 77]. Interestingly, *BRD1* (OMIM*604,589) encodes for a scaffold protein implicated in chromatin remodeling through histone H3K14 acetylation [39]; recently, *BRD1* deletion in patients with PMS was associated with a disruption of histone acetylation processes followed by DNA hypermethylation, supporting its crucial role as an epigenetic regulator [84]. *BRD1* is highly expressed in the brain, it is actively involved in neurodevelopment, and the haploinsufficient *Brd1* +/− mice exhibit different neurochemistry and altered synaptic morphology, leading to behavioral and cognitive impairment [85, 86]. Furthermore, *Brd1*^+/−^ female mice show decreased level of cortical serotonin and of striatal dopamine, leading to depression-like behaviors in the presence of environmental risk factors (e.g. sex and stress) [87, 88]. Indeed *BRD1* gene variants have been consistently associated with schizophrenia and bipolar disorder [89–91]. The DNA methylation and the metabolic profile of PMS patients carrying deletions encompassing *BRD1* are significantly different from those of patients carrying smaller deletions and are clearly altered (e.g. reduced energy production, decreased adaptation to metabolic conditions, and an abnormal reaction to hormones and cytokines), affecting also the expression of genes located outside the chromosome 22q deleted region and in other chromosomes [84, 92]. Hence epigenetic alterations resulting from *BRD1*-spanning deletions could well contribute additively or synergically to increase phenotypic severity in combination with *SHANK3* and with other genes involved in language delay and in other phenotypes, like seizures [93] and coincidence of an infectious disease at behavioral symptom onset (see Results).

### Motor development and function

Delayed motor development resulting in impaired motor skills (fine more than gross) is almost consistently present in PMS [1–3]**.** Larger deletions have been previously associated with greater developmental delay and motor impairment [16, 19, 42, 43, 94]. The majority of our patients with deletion sizes inferior to 3 Mb display normal motor development timing (Table 5). In contrast, almost all patients with deletions greater than 3 Mb exhibit delayed motor milestones (Table 5). In our cohort, the 3,039 kb maximum likelihood threshold best discriminating patients with normal vs delayed motor development timing falls into the long non-coding RNA (LncRNA) *EPIC1* (Fig. 2, Suppl. Figure 2), a well-known cancer-promoting lncRNA able to stimulate cell proliferation and inhibit apoptosis by binding to MYC [95]. In vitro, *EPIC1* expression and MYC binding preserve neuronal cells from oxidative damage [96]. Currently available evidence could potentially support roles of *EPIC1* in other features associated with PMS, such as cognitive regression and macrocephaly, more than delayed motor development. Instead, a relevant role may be more likely for *TBC1D22A*, the first coding gene located upstream of the 3,039 kb threshold for delayed motor development timing (Table 5, Fig. 2, Suppl. Figure 2). *TBC1D22A* is expressed in brain and encodes an important regulator of cell surface trafficking for G-protein-coupled receptors, probably acting as a GTPase-activating protein for Rab family protein [97]. Interestingly, this gene has been identified as a recessive cause of febrile seizures plus [98] and its locus has been linked to risk of seizures in PMS [99]. It also frequently spans interstitial deletions in several patients with SHANK-unrelated PMS [36, 37]. Finally, CpG islands regulating *TBC1D22A* expression are hypermethylated in patients with bipolar disorder (BD) and hypomethylated in patients with SCZ [100]. Collectively, these results point strongly toward *TBC1D22A* as playing a pivotal role in motor development timing and, more broadly, in the structure and function of CNS neural networks, also in terms of excitation/inhibition imbalance. In addition to deletion size, it will be interesting to search for possible epigenetic modifications in the non-deleted allele in patients with haploinsufficient regions encompassing this gene.

Gait and muscle strength are often impaired in PMS [1–4, 46, 101]. They are also significantly affected by deletion length, as documented in a previous [94] and in the present study (Table 3). For example, lack of autonomous walking is observed in our sample only among carriers of deletions greater than 5 Mb (Table 5). Based on our analysis, both these functions are affected by genes located at or just beyond a 5,013 kb threshold, with the most likely candidates represented by *ATXN10* and *FBLN1* (Fig. 2, Suppl. Fig. S3). The *ATXN10* gene is highly expressed in the brain especially during gestation and its expression decreases after birth, consistently with a crucial role in CNS development [39]. Initially the expansion of a pentanucleotide ATTCT repeat located in intron 9 has been identified as the cause of spinocerebellar ataxia-10 (SCA-10) [102]. More recently, a broader morphogenetic role has been recognized [103] and *ATXN10* haploinsufficiency has been proposed as a possible contributor to the neurodevelopmental phenotype of 22q13.31 microdeletion syndrome, caused by interstitial microdeletions involving *ATXN10* while sparing *SHANK3* [36, 104, 105]. Previous studies have proposed that, in addition to altered neurodevelopment due to both *ATXN10* and *FBLN1* haploinsufficiency, further contributions to gait deficits in patients with deleted *FBLN1* may stem from hand/feet dysmorphisms (large or fleshy hands), as this gene has been conclusively associated with Synpolydactyly-2 (OMIM n. 608,180) [104, 105].

In addition to this first threshold detecting *ATXN10* and *FBLN1*, a second threshold located at 7,363 kb points toward *MPPED1* and *SCUBE1* as additional contributors to reduced muscle strength (Table 5, Fig. 2, Suppl. Fig. S3B). *MPPED1* (metallo-phosphoesterase domain containing protein 1) is highly expressed in the human fetal brain [105] and reduced expression yields cognitive impairment in ovariectomized mice [106]. It modulates the development and function of neocortical and hippocampal neurons through its metallo-phosphodiesterase activity [107]. *SCUBE1* (Signal Peptide-CUB-EGF Domain-Containing Protein 1) is a morphogenetic gene expressed in different tissues, including the brain, kidney, platelets and endothelial cells [108]. Homozygous *Scube1*^*Δcub/Δcub*^mice lacking the CR and CUB domain display acrania, exencephaly, and death shortly after birth demonstrating the major role of this gene in early craniofacial development [109]. Rare de novo disruptive *SCUBE1* missense variants have been found in patients with obsessive–compulsive disorder [110].

While the first threshold detects strong candidate genes supported by multiple converging lines of evidence, to our knowledge this is the first study suggesting a possible association between the latter chromosomal region and reduced muscle strength. Given our limited sample size, despite reaching statistical significance (Table 3), this finding should be viewed especially with caution, until replicated in additional cohorts of PMS patients.

### Social cognition (eye contact, exchange gesture, joint attention)

Autism is frequently diagnosed among PMS patients, with prevalence estimates ranging from less than 30% to more than 80%, possibly higher among *SHANK3* mutation carriers and in patients with very small deletions [1–4, 29–34]. Compared with prior literature, rates of ASD are relatively low both in our overall sample (28.6%) [46] and in our patients carrying chromosome 22q deletions (25.4%) (Table 1). The partially overlapping clinical features of profound intellectual disability (ID) and severe ASD (for example, intense stereotypic behaviors are often present in both conditions), the use of ADOS scores instead of clinical DSM-5 criteria as primary measure to define the presence of ASD (ADOS is often positive when administered to individuals with profound ID), and an insufficient focus on the psychopathological foundations distinguishing ID from ASD (cognitive development congruent with an earlier age in ID vs primary interest in objects at the expense of social cognition and interactions in ASD) could explain the variability in ASD prevalence estimates reported in different studies [46]. Hence, in our genotype–phenotype correlation analysis, rather than using a categorical diagnostic approach, we focused on eye contact, reciprocal object exchange gesture, and joint attention, as elicited and observed by the clinician visiting each PMS patient. Eye contact, exchange gesture, and joint attention in the present context should not be viewed as a “proxy” for an ASD diagnosis, because they can be variably compromised also in individuals with profound Intellectual Disability who do not deserve a primary or co-morbid ASD diagnosis. This likely explains the apparent discrepancy between relatively low rates of an ASD diagnosis (Table 1) and higher numbers of patients displaying partial or severe deficits in these three parameters (Table 6).

These three social behaviors were all significantly correlated with deletion size and all converged upon the same threshold located at 3,282 kb (Tables 3 and 6), almost superimposable to the threshold located at 3,039 kb critical for motor development timing (Table 3). Deletions smaller than 3 Mb show a relatively even distribution, possibly with a slight predominance of normal vs dysfunctional phenotypes (Table 6). Instead, the majority of patients carrying deletions larger than 3 Mb show no exchange gesture and joint attention, while eye contact becomes mostly inconsistent (Table 6). This threshold again falls within the *TBC1D22A* gene (Fig. 2, Suppl. Fig. S4), already discussed in reference to motor developmental delay (see above). This consistency among different behavioral parameters interconnected with social cognition is indeed reassuring and supports the validity of this finding*.*

Apparently less penetrant as compared to the first threshold is a second threshold located at 4,743 kb, reaching nominal significance only for joint attention (Tables 3 and 6). We cannot exclude this threshold may also apply to eye contact and exchange gesture, but statistical power may be insufficient in our sample to detect this signal. This second threshold overlaps with the one identified for renal abnormalities (Suppl. Fig S7B). It falls within a cluster of long non-coding RNAs and miRNAs, but it may also point again at *ATXN10* and/or *FBLN1*, which are located only 260 and 500 kb upstream (see below).

### Infectious disease at onset of behavioral manifestations

In approximately half of our PMS patients, who do not display severe muscle hypotonia soon after birth, parents reported noticing initial motor and behavioral manifestations of PMS in concomitance with an infectious episode, usually an ear infection, a bronchitis or an infection of the upper airways [46]. Furthermore, patients with PMS frequently exhibit immune dysfunction, fostering recurrent infections, as well as asthma, food allergies, and atopic dermatitis [3, 7, 111, 112]. We identified two peaks related to dysimmunity. The first is located at 253 kb and falls next to two immune-relevant loci, namely *KLHDC7B* and its lncRNA *KLHDC7B-DT*, as well as *CIMAP1B* (Fig. 2, Suppl. Fig. S5). *KLHDC7B-DT* has been identified as one of the lncRNAs most contributing to lupus nephritic activity, acting as a key regulator of interferon-mediated processes and B-cell adaptive immunity [113]. A reduced expression of the same lncRNA and of *KLHDC7B*, as would be predicted to occur in the presence of haploinsufficiency, can result in increased cellular migration and invasion, as well as resistance to apoptosis in breast cancer cells [114]. Finally, *KLHDC7B* is one of only eight TNF family member genes which contribute to the prognostic risk score for adenopancreatic carcinoma, by determining the immunomodulatory response of the host to cancer [115]. On the other hand, the expression of *CIMAP1B* is correlated with M1 macrophage infiltration affecting placental development and the immunoregulation of fetal growth restriction [116]. This gene is highly expressed in the brain [116], and was also found implicated in glioma proliferation and apoptosis through the JAK/STAT pathway, critical for immune regulation processes [117]. Most importantly, it is strongly associated with T cell pathology in multiple sclerosis and significantly downregulated in T_H_17 cells during the course of experimental autoimmune encephalomyelitis [118]. Hence, *CIMAP1B* haploinsufficiency could well contribute to an aggressive neuroinflammation in the CNS.

The second threshold, located at 1,059 kb, fully overlaps with the second threshold concerning verbal language, as discussed above, and falls within the *BRD1* gene (Fig. 2, Suppl. Figure 1B). By acting as an epigenetic regulator, *BRD1* can seemingly produce chromatin changes significantly affecting not only neurodevelopment, but also immune function. *BRD1* has been identified as a key gene in promoting CD8 expression in developing thymocytes evolving toward the NK phenotype [119]. It is the strongest *in silico* candidate to explain immune infiltration in osteoarthritis [120]. Its downregulation activates NK and CD8 T cells to transition from inert to more active and cytotoxic phenotypes [121]: this effect may indeed be helpful in enhancing the efficacy of immunotherapy for various forms of infiltrating tumors [121], but could instead promote excessive neuroinflammation in the PMS brain.

Collectively, the evidence summarized above points toward *KLHDC7B*, *KLHDC7B-DT*, and especially *CIMAP1B* and *BRD1* as strong candidates for dysimmunity in PMS, outlining a plausible explanation for the worsening of behavioral and motor deficits coincident with infections in early infancy, as reported by the parents of many children with deletions involving these genes. In the most plausible scenario, peripheral immune activation would reverberate to the CNS, sparking central neuroinflammation ultimately leading to excessive synaptic pruning by activated microglia, responsible for motor and cognitive regression [122, 123].

### Structural abnormalities found in brain MRI

Abnormal brain MRI have been extensively reported in patients with PMS, both in *SHANK3*-related and in *SHANK3*-unrelated PMS [3, 36]. Reduced myelination, thin corpus callosum, global atrophy of white matter, brain asymmetry, reduced myelination and hypoplasia of the cerebellar vermis have been frequently reported [7, 8, 77]. Positron emission study (PET) showed a localized dysfunction of the left temporal pole and significant amygdala hypoperfusion in eight children with PMS, with normal structural brain MRI o just with thinning of the corpus callosum [8]. In our sample, 48/60 (80%) of PMS patients display at least one structural abnormality on brain MRI, while 30/60 (50%) display two or more [46] (Table 7; for specific brain anomalies see Suppl. Table S1). In the present study, three thresholds located at 451 kb, 833 kb and 5,024 kb from the 22q telomeric end were found to reach statistical significance (Table 3, Fig. 2). The 451 kb threshold falls into the first intron of the *PPP6R2* gene, just telomeric to *DENND6B* (Suppl. Fig. S6A). Both these genes do not appear as strong candidates to yield structural anomalies in the CNS, although both are expressed in the brain and cannot be entirely excluded. The morphogenetic gene *PLXNB2* (OMIM*604,263), located immediately centromeric to *DENND6B* and only 50 kb upstream of this first threshold, represents a much stronger candidate (Suppl. Fig. S6A). *PLXNB2* exerts multiple roles in cerebral, cerebellar, and kidney development [39]. It encodes for one of the Plexins, large transmembrane cell surface receptors able to control cell migration and axon guidance by binding semaphorins. It is involved in the closure of the neural tube [124], in the proliferation and migration of cerebellar granule cell precursors, in cell migration and differentiation during corticogenesis, and in the formation of excitatory synapses in hippocampal neurons [10, 124–126]. In addition to the CNS, also the developing kidney expresses *PLXNB2*, and *PLXNB2*-/- mice display kidney and urinary tract malformations [126]. In fact, Plxnb2 binds Sema4C and spurs the branching of the ureteric epithelium [127]. Hence, *PLXNB2* has already been proposed as a strong candidate gene for brain and cerebellar MRI phenotypes in PMS [10], as well as for kidney disorders in patients with 22q13 deletion syndrome [128]. Our results are thus compatible with the proposed association of *PLXNB2*, at least with brain and cerebellar imaging anomalies.

The second threshold is located at 833 kb and the first gene about 50 kb centromeric to this peak is *PIM3* (Suppl. Fig. S6B), which encodes for a serine/threonine kinase able to prevent apoptosis and to promote cell survival and protein translation [129]. In vivo, this gene is expressed in several tissues, including brain, and plays a crucial role in the self-renewal of embryonic stem cells, stimulating cell proliferation [130]. In rodent brain, *PIM3* is induced in the hippocampus in response to stimuli that elicit long-term potentiation [131]. Reduced *PIM3* gene expression can reduce proliferation both in normal and tumor cells [129, 130].

Moving 42.3 kb further upstream from *PIM3* is *ALG12* (Suppl. Fig. S6B), another potential candidate gene [36, 37]. *ALG12* encodes for an α6-mannosyltransferase and autosomal recessive biallelic inactivation of this gene produces *Congenital Disorder of Glycosylation, Type Ig* (ALG12-CDG) [132]. Compound heterozygous disruptive variants in *ALG12* have been reported in two patients with hypoplasia of the cerebrum, brain stem and cerebellar vermis [125]. The inactivation of a single allele should not be sufficient to yield brain structural anomalies in PMS patients with chromosome 22q13 deletions and the co-existence of a disruptive mutation located in the non-deleted allele is predicted to be a rare event unable to drive the statistical signal we detect in our sample. Conceivably, if the second threshold is not entirely due to *PIM3*, gene x gene interactions involving *ALG12* or contributions by low-function genetic or epigenetic variants may warrant further investigations.

Finally, the third peak is practically superimposable to the threshold involving *ATXN10* in gait, muscle strength and joint attention (Suppl. Fig. S6C), as described above. This result further points toward the potential relevance of *ATXN10* haploinsufficiency in shaping the PMS phenotype. However, a missense mutation, p.(Cys397Phe) in fibulin-1 *(FBLN1)* has been detected in three affected siblings exhibiting brain atrophy and compression of spinal cord on MRI [133]. Hence, once again it is not possible to exclude *FBLN1* contributions also in reference to brain morphology.

Overall, as many as 18/20 (90.0%) patients carrying deletions exceeding 5 Mb display two or more structural abnormalities (Table 7). These patients carry deletions encompassing all the candidate genes discussed above (*PIM3*, *PLXNB2*, *ALG12*, *ATXN10*, and *FBLN1*), while patients carrying larger deletions also suffer from deficits produced by additional known pathogenic genes, like *TCF20* [134–136]. Structural CNS abnormalities are thus likely to result from complex gene x gene, and gene x environment interactions involving multiple major loci located in chr22q13 and involved in brain macroanatomy.

### Renal abnormalities

Up to 40% of patients with PMS have malformations involving the kidneys or the upper urinary tracts, including renal cysts, renal hypoplasia or agenesis, hydronephrosis, vesicoureteral reflux, kidney dysplasia, horseshoe kidneys, and pyelectasis have been described in different genotype phenotype studies [3, 8, 46, 104, 128, 137]. Large deletions have been linked to renal abnormalities, and *SHANK3* seems to play no role in urinary tract dysfunction [4, 14, 137]. In prior studies, the genes *UPK3A, FBLN1, ZBED4, WNT7B, SCUBE1 RABL2B, PNPLA3, PLXNB2,* and *CELSR1* have been proposed as possibly associated with kidney disorders [14, 104, 128, 137]. In our sample, the prevalence of malformations of the kidney and of the upper urinary tract, or the presence of kidney stones increased considerably with a deletion size greater than 5 Mb (Table 7). We identify two maximum likelihood thresholds and both fall in gene-poor regions, enriched in non-coding transcripts, such as EPIC1, PRR34, MIRLET7BHG, and a cluster of antisense RNAs (Suppl. Figures 7 A and 7B). Genetic variants of the MIRLET7BHG short non-coding RNA (miR) have been associated with cancer [138] and polycystic ovarian syndrome[139]. In tissues from the prefrontal cortex of the brain of SARS-CoV-2 infected patients, *MIRLET7BHG* was found to be upregulated in excitatory neurons [140]. The second threshold may also involve *WNT7B*, in line with previous suggestive evidence [128, 137]. This gene is a member of the WNT gene family, encoding secreted signaling proteins implicated in oncogenesis and in developmental processes, including regulation of cell fate and patterning during embryogenesis; among members of the human WNT family, this gene product is most similar to the WNT7A protein, and is highly expressed in the kidney and urinary tract, where it exerts prominent developmental roles [141]. *WNT7B* thus appears an appealing candidate for contributions to this phenotype, in conjunction with other urinary morphogenetic genes, like *PLXN2B, UPK3A, FBLN1,* and *CELSR1*. However, while coding genes have received most attention [128, 137], our results indicate that the contribution of non-coding RNAs to renal and urinary tract malformations indeed deserve further scrutiny.

### Dysmorphisms

PMS patients can exhibit several dysmorphic features, such as large fleshy hands, bulbous nose, long eyelashes, ear malformations, hypoplastic or dysplastic nails, full lips, pointed chin, dolichocephaly, and microcephaly/macrocephaly [1–3, 94]. In our sample, deletions ≥ 2 Mb were clearly associated with greater incidence and severity of dysmorphic features both minor and major (Table 7). Our maximum likelihood threshold indicates *TAFA5* (also named *FAM19A5*) as the strongest candidate (Fig. 2, Suppl. Fig. S8). This gene is involved in the generation of neural stem cells and oligodendrocyte precursor cells, and acts as a regulator for immune and nervous cells both during development and in response to pathological conditions [142]. It was linked to depressive symptoms, neuroinflammation, neurodegeneration, and cognitive impairment both in humans and mice [143–145]. However, *TAFA5*/*FAM19A5* also inhibits RANKL-induced osteoclast differentiation essential for bone metabolism and homeostasis [146]. While contributions to CNS dysfunction cannot be excluded, this peripheral function as a modulator of osteoclastogenesis and bone remodelling may explain an association with dysmorphic features.

### Lifetime diagnosis of bipolar disorder

*SHANK3* deletion appears to be sufficient in enhancing the risk of co-morbid bipolar disorder. This result is in line with previous evidence documenting greater liability toward psychiatric comorbidities (bipolar and unipolar affective disorder, catatonia) in carriers of small deletions and *SHANK3* point mutations, compared to carriers of large deletions who suffer from more severe cognitive and motor impairment [22–25]. Conceivably, these comorbidities could occur primarily in the presence of a familial genetic liability toward affective disorders, whose penetrance may be enhanced by the *de novo* haploinsufficiency of *SHANK3*, but this hypothesis remains to be conclusively demonstrated.

### Prediction of functional impact for threshold-adjacent genes

To further test whether the heterozygous deletion of the proposed candidates gene can affect the clinical phenotype, in addition to performing a thorough literature review, we also annotated the most promising candidate genes with computational damage prediction metrics and with empirical data derived from the blood transcriptomic study by Breen et al., [70], who contrasted PMS patients with large vs small deletions (Table 9). Both pLI [67] and LOEUF [68] support a very limited number of our candidate genes, especially *BRD1* and *FBLN1* (Table 9). LOEUF, but not pLI, confirms the known pathomorphic gene *CELSR1* and detects also *PLXNB2* as a constrained gene (Table 9). This result is not surprising, because these metrics are sensitive to genetic constraint, i.e. they detect pathogenic disruptive mutations which severely impair fitness [147]. In our context, both pLI and LOEUF display at least two major limitations: (a) their prediction of genetic constraint measures the selective pressure exerted by the disruption of single genes, without taking into account the complex interactions among genes spanning the deleted region in large copy number variants (CNVs) and between these deleted genes and many other genes located in non-deleted chromosomes [70]; (b) in large chromosome 22q13 terminal deletions, while *SHANK3* clearly represents the primary *pathogenic* gene, other genes may well play *pathomorphic* roles in shaping the presence/absence, time course, and severity of specific clinical signs and symptoms, even if they are not “disease causing” *per se*. Finally, the pLI is endowed with several known pitfalls and should be interpreted with caution especially when negative [71]. A glaring example is represented here by *CELSR1*, an established disease modifier associated with lymphedema [13] displaying a pLI of 0 according to gnomAD v4.1.0 (Table 9).

To circumvent these limitations, (a) we employed the pHaplo statistics, an ensemble machine-learning score designed to predict the probability of gene dosage sensitivity specifically in CNVs [69]; and (b) we sought gene dosage transcriptional effects in the blood transcriptomic data set recently produced contrasting PMS patients carrying large vs small deletion [70]. Using these approaches, all the candidate genes proposed in the present study either yield a pHaplo score ≥ 0.86 or are dosage-sensitive at the transcriptional level, except for *DENND6B*, *UPK3A*, and *MPPED1,* which are not expressed in blood. This demonstrates that the majority of the candidate genes we identified are indeed dosage-sensitive at the transcriptional level and can be predicted to exert functional effects when in the context of a large deletion, though not necessarily producing severe genetic constraint when haploinsufficient at the single gene level.

The fundamental distinction that needs to be made between “pathogenic” and “pathomorphic” genes in CNV-associated genetic syndromes, the complexity of gene–gene interactions in determining the functional consequences of large CNVs [70], as well as the results displayed in Table 9, in our opinion all raise caution into excluding *a priori* candidate genes identified performing empirical genotype–phenotype correlations, based solely on computational predictions. Also the existence of rare autosomal recessive conditions may justify the exclusion of disease-causing pathogenic roles for haploinsufficiency at a given locus, but not of pathomorphic contributions to the clinical picture. Specifically, the largest chromosome 22q13.3 deleted region in PMS contains 13 genes with well-proven roles in autosomal recessive conditions [46], with the possible addition of *SCO2* and recently of *PLXNB2* [148]. Still, especially for morphogenetic genes like *PLXNB2*, our results suggest that their haploinsufficiency, in conjunction with *SHANK3* haploinsufficiency, may well produce pathomorphic changes specifically in the context of PMS (Table 9). Nonetheless, the present study is limited by sample size (see below), and meta-analyses combining multiple samples of directly-ascertained patients will indeed largely refine the gene list presented here and add other relevant genes, hopefully providing evidence convergent with results from registry-based studies.

### Strengths and limitations

The main limitation of this study is its sample size, which is statistically small, though substantial when considering that PMS is a rare disorder, all patients were directly ascertained [46], and the vast majority of similar studies published to date have a smaller sample size [3, 14, 35, 42, 44]. Instead, studies collecting data from registries can reach many more patients [40], at the expense of reliability and precision, as investigators have no control over information provided by family members and no direct interactions with the patient. In the latter setting, some clinical phenotypes cannot be explored, while others require caution because parents may overlook or overemphasize some behaviors/traits. In our opinion, direct ascertainment can yield the most reliable results and allow to explore a broader array of phenotypes. Nonetheless, we acknowledge the relatively large regions of association stemming from our limited statistical power (Table 3). Furthermore, some results are sufficiently clear-cut, but for other phenotypic features additional thresholds may have been missed. Ultimately, merging and meta-analyzing data from several direct-ascertainment studies may represent the best strategy to enhance statistical power while retaining maximum reliability.

Furthermore, most 22q deletions carried by our patients are simple rearrangements, but we cannot exclude phenotypic effects by at least two additional mechanisms which were not investigated here, namely (a) an abnormal epigenetic modulation of gene expression in the residual allele, and (b) complex chromosomal rearrangements and uniparental disomy yielding a global alteration of the three-dimensional chromatin architecture of chromosome 22, as previously described in a subset of patients with 22q deletions and PMS [149, 150].

## Conclusions

The present study, in conjunction with the recently published clinical phenotyping of our PMS sample [46], provides new evidence supporting a significant correlation between terminal deletion size in chr 22q11.3 and several phenotypes including verbal language, motor development timing, gait, muscle strength, social cognition (i.e. eye contact, reciprocal object exchange gesture, and joint attention), infectious diseases coincident with the behavioral onset of PMS, brain structural abnormalities on MRI, renal and upper urinary tract anomalies, dysmorphisms, and comorbidity with bipolar disorder. We find several recurrent thresholds pointing at strong candidate genes possibly involved in one or in several phenotypes through their important role in neurodevelopment (*BRD1*, *TBC1D22A*, *ATXN10* and/or *FBLN1*), and morphogenesis (*PLXNB2, TAFA5*). Only for renal malformations, the two maximum likelihood thresholds both point toward long non-coding RNAs and antisense RNAs, whose functional impact deserves further investigation. Larger samples will be able to refine these results and to detect additional phenotypic characteristics influenced by genes other than *SHANK3*. Other genes involved in very large deletions yield serious consequences on brain morphology, motor and cognitive function, a well-known example consisting in *TCF20* [134–136], located at 8,577 kb from the telomeric end (Table 2). Our results should not be interpreted as overlooking the role of these more centromeric genes, rather as demonstrating that smaller deletions can be equally disruptive because they already span genes closer to *SHANK3,* which play a critical role in multiple clinically-relevant phenotypes, including CNS structure and function. This line of investigation is important, because it can significantly improve the clinical management of PMS patients by conferring valuable prognostic information to clinicians on the basis of deletion size already at the time of genetic diagnosis. The replication and consistency among studies assessing different cohorts of clinically-characterized PMS patients carrying chr 22q13 terminal deletions, as well as interstitial deletions sparing *SHANK3*, will be instrumental in reliably mapping the precise landscape of genotype–phenotype correlations in Phelan-McDermid syndrome. Future investigations will also need to address additional mechanisms able to contribute to the heterogeneity of the clinical phenotype, in conjunction with deletion size.

## Supplementary Information

Supplementary Material 1.

## Data Availability

The datasets used and/or analyzed during the current study are available from the corresponding author on reasonable request.
