# Supplementary material for: Chromosome 22q13 terminal deletion size is associated with relevant clinical features in a sample of 63 Italian patients with Phelan-McDermid syndrome
Source: J Neurodev Disord. 2026 May 26;18:53. doi: 10.1186/s11689-026-09708-x (PMC13584495; doi:10.1186/s11689-026-09708-x)
Supplement: Supplementary file 1 [file 11689_2026_9708_MOESM1_ESM.docx]

**Supplementary Material**

**Chromosome 22q13 terminal deletion size is associated with relevant clinical features in a sample of 63 Italian patients with Phelan-McDermid syndrome.**

**Laura Sandoni^1^, Fethia Chehbani^1^, Lisa Asta^1^, Michela Camia^1^, Arianna Ricciardello^2^, Pasquale Tomaiuolo^3^, Francesca Cucinotta^4^, Laura Turriziani^5^, Maria Boncoddo^6^, Fabiana Bellomo^7^, Marco Baccarin^8^, Chiara Picinelli^3^, Paola Castronovo^3^, Roberto Sacco^9^, Carla Lintas,^10,11^ Ignazio Stefano Piras^12^, Francesco Pelagatti^1^, Federico Banchelli^13^, Riccardo Cuoghi Costantini^13^, Roberto D'Amico^13,14^, Antonio M. Persico^1,15^**

^1^Department of Biomedical, Metabolic and Neural Sciences, University of Modena and Reggio Emilia

^2^Cantonal Psychiatric Clinic, Cantonal Socio-psychiatric Organization (O.S.C.), Repubblica e Cantone Ticino, Mendrisio, Switzerland

^3^Mafalda Luce Center for Pervasive Developmental Disorders, Milan, Italy

^4^IRCCS Centro Neurolesi "Bonino-Pulejo", Messina, Italy

^5^Center for Autism “Dopo di noi”, Barcellona Pozzo di Gotto (Messina), Italy

^6^Institute for Biomedical Research and Innovation (IRIB), National Research Council (CNR), Messina, Italy

^7^Child Neuropsychiatry Unit, “G. Martino” University Hospital, Messina, Italy

^8^Medical Genetics Service (EOLAB), Ente Ospedaliero Cantonale, Bellinzona, Switzerland

^9^Service for Neurodevelopmental Disorders & Laboratory of Molecular Psychiatry and Neurogenetics, University “Campus Bio-Medico”, Rome, Italy

^10^Research Unit of Medical Genetics, Department of Medicine, University Campus-Biomedico of Rome, I-00128 Rome, Italy;

^11^Operative Research Unit of Medical Genetics, Fondazione Policlinico Universitario Campus Bio-Medico, I-00128 Rome, Italy

^12^Division of Early Detection and Prevention, The Translational Genomics Research Institute, Phoenix, AZ 85004, USA

^13^Unit of Statistical and Methodological Support to Clinical Research, Modena Univ. Hospital, Modena, Italy

^14^Department of Medical and Surgical Sciences, University of Modena and Reggio Emilia

^15^Child & Adolescent Neuropsychiatry Program, Modena University Hospital, Modena, Italy.

***Correspondence:** antonio.persico@unimore.it.

ORCID numbers: Laura Sandoni 0009-0005-9017-9714, Fethia Chehbani 0000-0002-6649-5048, Lisa Asta 0000-0003-1619-237X, Michela Camia 0000-0002-8753-9839, Arianna Ricciardello 0000-0001-9926-0627, Pasquale Tomaiuolo 0000-0002-1540-3924, Francesca Cucinotta 0000-0001-8477-7826, Laura Turriziani 0009-0005-7173-2879, Maria Boncoddo 0009-0003-8817-7176, Roberto Sacco 0000-0003-3129-5545, Ignazio Stefano Piras 0000-0003-4024-3368, Federico Banchelli 0000-0003-0499-2505, Riccardo Cuoghi Costantini 0000-0002-9116-5198, Roberto D'Amico 0000-0002-3211-6687, Antonio M. Persico: 0000-0001-8910-4479.

**Suppl. Table S1.** Distribution of brain structural abnormalities on MRI by deletion size category (total N=60).

| **Deletion size** | **Genes** | **Normal** | **Thin corpus callosum** | **Dysmorphic isthmus of the corpus callosum** | **Lateral**  **ventricular**  **dilation** | **Perinatal lesions in white matter** | **Delayed myelination** | **Mega cisterna magna** | **Others** | **Multiple anomalies**  **(2 or more)** |
| --- | --- | --- | --- | --- | --- | --- | --- | --- | --- | --- |
|  |  |  |  |  |  |  |  |  |  |  |
|  |  |  |  |  |  |  |  |  |  |  |
| <132 kb | *RABL2B* | **8 (50%)** | 1 (6.25%) | 1 (6.25%) | 2 (12.5%) | 1 (6.25%) | 0 (0%) | 0 (0%) | 1 (6.25%) | 2 (12.5%) |
|  | *SHANK3* |  |  |  |  |  |  |  |  |  |
| 132 - 990 kb | *ARSA* | **2 (25%)** | 0 (0%) | 0 (0%) | 1 (12.5%) | 0 (0%) | 0 (0%) | 0 (0%) | 1 (12.5%) | **4 (50%)** |
|  | *MAPK8IP2* |  |  |  |  |  |  |  |  |  |
|  | *CHKB* |  |  |  |  |  |  |  |  |  |
|  | *CIMAP1B* |  |  |  |  |  |  |  |  |  |
|  | *TYMP* |  |  |  |  |  |  |  |  |  |
|  | *SCO2* |  |  |  |  |  |  |  |  |  |
|  | *SBF1* |  |  |  |  |  |  |  |  |  |
|  | *PLXNB2* |  |  |  |  |  |  |  |  |  |
|  | *TUBGCP6* |  |  |  |  |  |  |  |  |  |
|  | *MLC1* |  |  |  |  |  |  |  |  |  |
|  | *ALG12* |  |  |  |  |  |  |  |  |  |
| 1-1.99 Mb | *BRD1* | 0 (0%) | 1 (16.7%) | 0 (0%) | 0 (0%) | 0 (0%) | 1 (16.67%) | 0 (0%) | 0 (0%) | **4 (66.7%)** |
| 2-2.99 Mb | *TAFA5* | 0 (0%) | 1 (33.3%) | 0 (0%) | 1 (33.3%) | 0 (0%) | 0 (0%) | 0 (0%) | 0 (0%) | **1 (33.3%)** |
| 3-3.99 Mb | *TBC1D22A* | 2 (50%) | 0 (0%) | 0 (0%) | 0 (0%) | 0 (0%) | 0 (0%) | 0 (0%) | 1 (25%) | **1 (25%)** |
| 4-4.99 Mb | *CELSR1* | 0 (0%) | 0 (0%) | 1 (33.3%) | 0 (0%) | 1 (33.3%) | 0 (0%) | 1 (33.3%) | 0 (0%) | **0 (0%)** |
|  | *TRMU* |  |  |  |  |  |  |  |  |  |
| 5-5.99 Mb | *ATXN10* | 0 (0%) | 1 (14.3%) | 0 (0%) | 0 (0%) | 0 (0%) | 0 (0%) | 0 (0%) | 1 (14.3%) | **5 (71.4%)** |
|  | *FBLN1* |  |  |  |  |  |  |  |  |  |
|  | *UPK3A* |  |  |  |  |  |  |  |  |  |
| 6-6.99 Mb | *PRR5* | 0 (0%) | 0 (0%) | 0 (0%) | 0 (0%) | 0 (0%) | 0 (0%) | 0 (0%) | 0 (0%) | **2 (100%)** |
|  | *PARVB* |  |  |  |  |  |  |  |  |  |
|  | *SAMM50* |  |  |  |  |  |  |  |  |  |
|  | *PNPLA3* |  |  |  |  |  |  |  |  |  |
| 7-7.99 Mb | *SULT4A1* | 0 (0%) | 0 (0%) | 0 (0%) | 0 (0%) | 0 (0%) | 0 (0%) | 0 (0%) | 0 (0%) | **5 (100%)** |
|  | *MPPED1* |  |  |  |  |  |  |  |  |  |
|  | *SCUBE1* |  |  |  |  |  |  |  |  |  |
|  | *BIK* |  |  |  |  |  |  |  |  |  |
| 8-8.99 Mb | *A4GALT* | 0 (0%) | 0 (0%) | 0 (0%) | 0 (0%) | 0 (0%) | 0 (0%) | 0 (0%) | 0 (0%) | **5 (100%)** |
|  | *CYB5R3* |  |  |  |  |  |  |  |  |  |
|  | *RNU12* |  |  |  |  |  |  |  |  |  |
|  | *TCF20* |  |  |  |  |  |  |  |  |  |
|  | *CYP2D6* |  |  |  |  |  |  |  |  |  |
|  | *NDUFA6* |  |  |  |  |  |  |  |  |  |
|  | *NAGA* |  |  |  |  |  |  |  |  |  |
|  | *TNFRSF13C* |  |  |  |  |  |  |  |  |  |
| >9 Mb | *SREBF2* | 0 (0%) | 0 (0%) | 0 (0%) | 0 (0%) | 0 (0%) | 0 (0%) | 0 (0%) | 0 (0%) | **1 (100%)** |

**Figure S1.** *Presence/absence of verbal expressive language*: genome browser view of the chromosomal segment immediately centromeric to (A) the maximum likelihood threshold located at 114 kb from the telomeric end of chromosome 22q, which falls within the *SHANK3* gene (red arrow), and (B) the second threshold located at 1,059 kb, within the *BRD1* gene. This second threshold also significantly influences the “presence/absence of infectious pathology at behavioral symptom onset”.

**Figure S2.** *Motor development timing*: chromosomal segment centromeric to the maximum likelihood threshold located at 3,039 kb from the telomeric end of chromosome 22q (red arrow). The maximum likelihood threshold falls within the “epigenetically induced MYC interacting lncRNA 1” (*EPIC1*), while the first expressed downstream gene is *TBC1D22A*.

**Figure S3.** *Gait and muscle strength*: chromosomal segment centromeric to (**A**) the maximum likelihood threshold for gait, falling within the *ATXN10* gene sequence (*FBLN1* is just centromeric). This threshold is practically superimposable to threshold n. 2 for muscle strength (5,065 and 5,013 kb, respectively); and (**B**) the best cut-off (threshold n. 1) for muscle strength located at 7,363 kb, inside the *MPPED1* gene sequence (*SCUBE1* is just centromeric).

**Figure S4.** *Eye contact, reciprocal object exchange gesture, joint attention:* chromosomal segment centromeric to the first threshold located at 3,282 kb from the telomeric end of chromosome 22q13. The first coding gene centromeric to this threshold is *TBC1D22A* gene, whereas the *EPIC1* lncRNA is immediately telomeric this threshold and is thus not depicted. Another threshold, recognized for joint attention only and located at 4,743 kb, coincides with the second threshold identified for kidney and upper urinary tract anomalies (see Suppl. Fig. S7B).

**Figure S5.** *Presence/absence of infectious pathology at behavioral symptom onset*: chromosomal segment centromeric to the first threshold located at 253 kb. The first expressed gene centromeric to this best cut-off is *CIMAP1B*. The second threshold for this phenotypic variable is located at 1,059 kb, within the *BRD1* gene (same as in Suppl. Fig. 1B).

**Figure S6.** *Brain anomalies on MRI*: chromosomal segment centromeric to (**A**) the first threshold located at 451 kb from the telomeric end of chromosome 22q (red arrow), which falls within *PPP6R2*. The first known morphogenetic gene is *PLXNB2*, located approximately 50 kb upstream; (**B**) the second threshold is located at 833 kb. The first expressed gene centromeric to this threshold is *PIM3*; (**C**) the third threshold falls within the *ATXN10* gene, similarly to thresholds for gait and muscle strength (see Suppl. Fig. S3A).

**Figure S7.** *Kidney and upper urinary tract anomalies*: chromosomal segment centromeric to (**A**) the first threshold, located at 2,953 kb, which identifies the *EPIC1* lncRNA, already detected for motor development timing (see Suppl. Fig. S2); and (**B**) the second threshold located at 4,743 kb from the telomeric end of chromosome 22q13. This region is enriched in antisense RNAs and in long non coding RNAs, like PRR34 and the highly conserved MIRLET7BHG.

**Figure S8.** *Dysmorphisms*: chromosomal segment centromeric to the threshold located at 1,860 kb from the telomere. The closest coding gene upstream of the threshold is *TAFA5*.

**Figure S9.** *Comorbid bipolar disorder*: the two thresholds both fall very close or within the *SHANK3* gene sequence.
